# NOTCH3 expression is linked to breast cancer seeding and distant metastasis

**DOI:** 10.1186/s13058-018-1020-0

**Published:** 2018-09-04

**Authors:** Alexey A. Leontovich, Mohammad Jalalirad, Jeffrey L. Salisbury, Lisa Mills, Candace Haddox, Mark Schroeder, Ann Tuma, Maria E. Guicciardi, Luca Zammataro, Mario W. Gambino, Angela Amato, Aldo Di Leonardo, James McCubrey, Carol A. Lange, Minetta Liu, Tufia Haddad, Matthew Goetz, Judy Boughey, Jann Sarkaria, Liewei Wang, James N. Ingle, Evanthia Galanis, Antonino B. D’Assoro

**Affiliations:** 1 Department of Biomedical Statistics and Informatics, Mayo Clinic College of Medicine, 200 First Street SW, Rochester, MN USA; 2 Department of Medical Oncology, Mayo Clinic College of Medicine, 200 First Street SW, Rochester, MN USA; 3 Department of Biochemistry and Molecular Biology, Mayo Clinic College of Medicine, 200 First Street SW, Rochester, MN USA; 4 Department of Molecular Medicine, Mayo Clinic College of Medicine, 200 First Street SW, Rochester, MN USA; 5 Department of Internal Medicine, Mayo Clinic College of Medicine, 200 First Street SW, Rochester, MN USA; 6 Department of Obstetrics, Gynecology, and Reproductive Sciences, Yale University School of Medicine, New Haven, CT USA; 7 Department of Cellular and Developmental Biology, University of Palermo, Palermo, Italy; 8 Department of Microbiology and Immunology, Brody School of Medicine, East Carolina University, Greenville, NC USA; 9 Department of Medicine and Pharmacology, University of Minnesota, Minneapolis, MN USA; 10 Department of Surgery, Mayo Clinic College of Medicine, 200 First Street SW, Rochester, MN USA

**Keywords:** Breast cancer, Metastasis, Chromosomal instability, Centrosome amplification, Tumor stemness

## Abstract

**Background:**

Development of distant metastases involves a complex multistep biological process termed the *invasion-metastasis cascade*, which includes dissemination of cancer cells from the primary tumor to secondary organs. NOTCH developmental signaling plays a critical role in promoting epithelial-to-mesenchymal transition, tumor stemness, and metastasis. Although all four NOTCH receptors show oncogenic properties, the unique role of each of these receptors in the sequential stepwise events that typify the invasion-metastasis cascade remains elusive.

**Methods:**

We have established metastatic xenografts expressing high endogenous levels of NOTCH3 using estrogen receptor alpha-positive (ERα^+^) MCF-7 breast cancer cells with constitutive active Raf-1/mitogen-associated protein kinase (MAPK) signaling (vMCF-7^Raf-1^) and MDA-MB-231 triple-negative breast cancer (TNBC) cells. The critical role of NOTCH3 in inducing an invasive phenotype and poor outcome was corroborated in unique TNBC cells resulting from a patient-derived brain metastasis (TNBC-M25) and in publicly available claudin-low breast tumor specimens collected from participants in the Molecular Taxonomy of Breast Cancer International Consortium database.

**Results:**

In this study, we identified an association between NOTCH3 expression and development of metastases in ERα^+^ and TNBC models. ERα^+^ breast tumor xenografts with a constitutive active Raf-1/MAPK signaling developed spontaneous lung metastases through the clonal expansion of cancer cells expressing a NOTCH3 reprogramming network. Abrogation of NOTCH3 expression significantly reduced the self-renewal and invasive capacity of ex vivo breast cancer cells, restoring a luminal CD44^low^/CD24^high^/ERα^high^ phenotype. Forced expression of the mitotic Aurora kinase A (AURKA), which promotes breast cancer metastases, failed to restore the invasive capacity of NOTCH3-null cells, demonstrating that NOTCH3 expression is required for an invasive phenotype. Likewise, pharmacologic inhibition of NOTCH signaling also impaired TNBC cell seeding and metastatic growth. Significantly, the role of aberrant NOTCH3 expression in promoting tumor self-renewal, invasiveness, and poor outcome was corroborated in unique TNBC cells from a patient-derived brain metastasis and in publicly available claudin-low breast tumor specimens.

**Conclusions:**

These findings demonstrate the key role of NOTCH3 oncogenic signaling in the genesis of breast cancer metastasis and provide a compelling preclinical rationale for the design of novel therapeutic strategies that will selectively target NOTCH3 to halt metastatic seeding and to improve the clinical outcomes of patients with breast cancer.

**Electronic supplementary material:**

The online version of this article (10.1186/s13058-018-1020-0) contains supplementary material, which is available to authorized users.

## Background

Breast cancer represents the second leading cause of cancer death among women worldwide [1]. Each year it is estimated that over 240,000 women in the United States will be diagnosed with breast cancer and that more than 40,000 will die of tumor relapse and metastatic dissemination to distant organs [2]. Although breast cancer research has been devoted largely to characterization of the molecular mechanisms responsible for tumor development, metastatic growth in secondary organs after surgical eradication of the primary tumor is responsible for poor outcomes [3]. For this reason, a better understanding of the molecular mechanisms leading to cancer cell seeding and metastatic growth is imperative to develop innovative therapies that will selectively target breast tumor metastasis-initiating cells (BT-MICs) and halt tumor progression.

Several studies have demonstrated that aberrant activation of mitogen-associated protein kinase (MAPK) oncogenic signaling induces drug resistance, development of distant metastases, and ultimately poor outcome of patients with breast cancer [4–6]. However, the molecular mechanisms by which the MAPK signaling pathway promotes cancer cells seeding and metastatic dissemination are poorly understood. It has been established that cross-talk between NOTCH and MAPK pathways in breast cancer correlates with early tumor relapse and poor overall survival [7], suggesting that NOTCH developmental signaling is a key mediator of MAPK-induced metastases. Canonical NOTCH signaling consists of four NOTCH receptors (NOTCH1–4) and their ligands (Delta-like 1, 3, and 4 and Jagged 1 and 2). All receptors are synthesized as a precursor form consisting of extracellular, transmembrane, and intracellular subunits [8]. In the most widely accepted model of NOTCH activation, ligand binding unfolds the negative regulatory region admitting the second cleavage through metalloproteinases of the ADAM family [9]. After this, γ-secretase complex performs an intramembrane cleavage releasing the NOTCH intracellular domain that translocates to the nucleus [10]. Following NOTCH activation, the hairy and enhancer of split (HES) family and the hairy-related transcription factor are expressed and in turn orchestrate the NOTCH-induced nuclear reprogramming [11, 12]. Aberrant activation of NOTCH oncogenic signaling promotes an invasive phenotype through activation of epithelial-to-mesenchymal transition (EMT) signaling [13]. Changes during EMT drive the transition from a polarized epithelial phenotype to an elongated fibroblastoid-like phenotype that typifies the morphology of highly metastatic cancer cells. These cancer cells exhibit a more invasive behavior characterized by downregulation of epithelial proteins (E-cadherin and claudin) responsible for cell adhesion and upregulation of mesenchymal proteins (N-cadherin and vimentin) involved in cell motility [14]. Several studies have established that breast cancer cells that undergo EMT acquire a CD44^high^/CD24^low^ cancer stemlike phenotype characterized by an increased capacity for tumor self-renewal, drug resistance, and high metastatic proclivity [15–17]. The discovery of breast tumor-initiating cells (BTICs) with a CD44^high^/CD24^low^ phenotype has been critical to elucidating the molecular mechanisms responsible for early recurrence and onset of distant metastases in advanced breast cancer. The correlation between aberrant activation of NOTCH/HES1 stemness signaling and tumor metastases has been revealed through a series of experimental investigations [18, 19]. Although all four NOTCH receptors can increase HES1 expression, whether a specific NOTCH receptor is mainly responsible for HES1 overexpression and transcriptional activity during the early stages of metastatic dissemination has not been established [20]. Recent findings propose that NOTCH signaling may promote the early onset of distant metastases through activation of C-X-C chemokine receptor type 4, a chemokine receptor that plays a key role in fostering cancer cell seeding to secondary organs [21, 22]. Although these studies show the redundant activity of NOTCH signaling, individual NOTCH receptors are likely to regulate breast cancer cells in unique ways; hence, it is essential to delineate the functional role for specific NOTCH receptors in driving tumor progression. Importantly, the NOTCH signaling pathway represents a powerful “druggable target” for cancer stemlike cells, which are known to be resistant to conventional chemotherapy and radiation but seem especially sensitive to inhibition of key stem cell pathways [23]. Several classes of investigational pan-NOTCH inhibitors have been developed that include γ-secretase inhibitors (GSIs) and humanized monoclonal antibodies against NOTCH receptors [24, 25]. Although GSIs have numerous substrates besides NOTCH receptors, the pharmacologic activity and toxicity of GSIs in vivo appears to be due largely to NOTCH inhibition [26]. GSIs have been administered to patients in phase I clinical trials, either as single agents or in combination with standard chemotherapy, with promising results [27, 28].

In this study, we demonstrated that NOTCH3 expression is linked to cancer cell seeding and development of breast cancer metastases. Using variant estrogen receptor alpha-positive (ERα^+^ MCF-7) breast tumor xenografts with constitutive active Raf-1/MAPK signaling (vMCF-7^Raf-1^), we showed that metastatic cancer cells display a clonal origin and increased expression of NOTCH3 that is required to induce self-renewal, stemness, and high invasive capacity. Significantly, forced expression of the mitotic Aurora kinase A (AURKA), which promotes stemness and breast cancer metastases [29], failed to restore the invasive capacity of NOTCH3-null vMCF-7^Raf-1^ cells, demonstrating that NOTCH3 oncogenic signaling is downstream of AURKA and is essential to inducing breast cancer cell invasiveness. The role of NOTCH3 expression in inducing a metastatic phenotype was corroborated in highly invasive MDA-MB-231 triple-negative breast cancer (TNBC) cells isolated from lung metastases. Moreover, we also demonstrated the clinical relevance of the NOTCH3 signaling pathway in promoting tumor invasiveness and poor outcome in unique patient-derived TNBC brain metastasis and publicly available claudin-low breast tumor specimens collected from participants of the Molecular Taxonomy of Breast Cancer International Consortium (METABRIC) database [30]. Taken together, these findings revealed the critical role of NOTCH3 oncogenic signaling in the genesis of breast cancer metastases and provided a compelling preclinical rationale for the design of novel therapeutic strategies that will selectively target the NOTCH3 signaling pathway to improve the clinical outcome of patients with advanced breast cancer.

## Methods

### Established breast cancer cell lines

The human breast cancer cell lines MCF-7 and MDA-MB-231 were obtained from the American Type Culture Collection (Mayo Clinic, Manassas, VA, USA). The MCF-7 cells overexpressing the Raf-1 oncoprotein were generated as previously described [29]. Human mammary epithelial cells (HMEC) were kindly provided by Wilma Lingle, PhD (Mayo Clinic). All cell lines were maintained in DMEM containing 5 mM glutamine, 1% penicillin/streptomycin, 20 μg/ml insulin (only for MCF-7 and their variants), and 10% FBS at 37 °C in a 5% CO_2_ atmosphere.

### Human breast cancer xenografts

Procedures established by the institutional animal care and use committee based on the National Institutes of Health Guidelines for the Care and Use of Laboratory Animals were followed for all experiments. Four-week-old nonovariectomized female NCR/Nu/Nu nude mice were anesthetized by exposure to 3% isoflurane, and five mice per group were given subcutaneous injections with 2 × 10^6^ MCF-7 or vMCF-7^∆Raf-1^ cancer cells suspended in 50 μl of 50% Matrigel (BD Biosciences, San Jose, CA, USA). Tumor localization and growth was monitored using an IVS imaging system (IVS, Coppell, TX, USA) from the ventral view 10 minutes after luciferin injection. After 12 weeks, mice were killed, and xenograft tumors were processed for histology and IHC analyses. Animals were examined every day, and body weight and primary tumor size were measured at least one or two times per week. Consistent distress and potential pain (> 1 day) were alleviated by killing the mice. If some of the animals were losing more than 10% of their body weight or if blood was consistently observed in the urine or around the genitals of the mice, the mice were appropriately killed. When typical signs of distress, including labored breathing and inactivity, were consistently observed for > 1 day, the animals were appropriately killed. When the primary tumor was > 2 cm, the animals were killed. Animals were killed using pentobarbital (100 mg/kg intraperitoneally) followed by cervical dislocation. The Mayo Clinic Institutional Animal Care and Use Committee approved this study (A00002634-17). Breast tumor xenografts and experimental lung metastases were established as previously described [29, 31]. To reestablish cultures from 1GX explants, primary tumors and metastatic lungs were excised from killed animals, minced using sterile scissors, and transferred to complete culture medium, and fibroblast-free tumor cell lines were established by serial passages in culture.

### Patient-derived TNBC cells

TNBC-M25 cells were isolated from a patient-derived brain metastasis TNBC xenograft model (EX170416) that was generated by the Breast Cancer Genome-Guided Therapy study (BEAUTY) in the Mayo Clinic (A17713) [32]. To establish cultured TNBC-M25 cells, patient-derived xenograft model EX170416 was excised from killed animals, minced using sterile scissors, and transferred to complete culture medium, and fibroblast-free TNBC-M25 cells were propagated in culture and used for this study.

### Immunoblot, immunofluorescence, and fluorescence-activated cell sorting assays

Immunoblot and immunofluorescence assays were performed as previously described [29]. Antibodies employed to perform these studies were as follows: centrin (20H5 kindly provided by Dr. Salisbury’s laboratory at the Mayo Clinic); ERα and pericentrin (Santa Cruz Biotechnology, Dallas, TX, USA); AURKA (Cell Signaling Technology, Danvers, MA, USA); CD44, CD24, NOTCH1, NOTCH2, and NOTCH3 (Abcam, Cambridge, MA, USA); and β-actin and α-tubulin (Sigma-Aldrich, St. Louis, MO, USA). Secondary antibodies were obtained from Molecular Probes (Eugene, OR, USA).

### Gene microarray analysis

Total RNA was extracted from basal-like CD24^−/low^ cells isolated by fluorescence-activated cell sorting (FACS) from vMCF-7^Raf-1^ 1GX cells as previously described [29], and mammospheres (MPS) were derived from vMCF-7^Raf-1^ 1GX-M cells using TRIzol reagent according to the manufacturer’s instructions (Life Technologies, Carlsbad, CA, USA). Total RNA (1 μg; A_260_/A_280_ ratio of 1.8–2.2) was used to probe for global genome expression employing Affymetrix U133 Plus 2.0 chips (Affymetrix, Santa Clara, CA, USA). Gene network and functional enrichment analysis was performed employing MetaCore software (GeneGo, St. Joseph, MI, USA). Two independent sets of experiments were performed. The raw data regarding the transcriptomic analysis can be accessed in the Gene Expression Omnibus database (http://www.ncbi.nlm.nih.gov/geo/info/linking.html).

### Cytogenetic and spectral karyotyping analysis

Cell harvest and metaphase slide preparation for routine cytogenetic and spectral karyotyping (SKY) analysis were performed as previously described [33]. Hybridization, wash, and detection of the human SKYPaint® probe (Applied Spectral Imaging, Carlsbad, CA, USA) were performed as recommended by the manufacturer. Image acquisition and spectral analysis of metaphase cells were achieved by using the SD200 SpectraCube™ Spectral Imaging System (Applied Spectral Imaging) mounted on a Zeiss Axioplan2 microscope (Carl Zeiss MicroImaging, Inc., Thornwood, NY, USA). Images were analyzed using HiSKY analysis software (Applied Spectral Imaging).

### Mammosphere formation

Human breast cancer cells were plated in ultralow attachment 24- and 96-well culture dishes in 100 μl of MammoCult™ medium (STEMCELL Technologies, Vancouver, BC, Canada). Medium was added every 2 days for a maximum of 8 days. MPS formation was recorded after 24 days through a digital camera (Nikon Instruments, Melville, NY, USA).

### Real-time apoptosis assay

MDA-MB-231 lung metastasis (LM) cells (*n* = 30,000) were plated in Costar 12-well plates (Corning Life Sciences, Oneonta, NY, USA) and incubated with YOYO-1 iodide. After 24 hours, cells were treated with 500 nM alisertib or 500 nM LY-411575 and incubated for additional 24 hours in the presence of YOYO-1 iodide. Apoptotic cells were quantified in real time using IncuCyte S3 (Essen BioScience, Ann Arbor, MI, USA). Experiments were performed in triplicate (± SD).

### Real-time invasion assay

Cancer cell invasion capacity was assessed using 24-well plate cell culture inserts equipped with a light-tight polyethylene terephthalate membrane (8-μm pore size, Corning® FluoroBlok™ 351152; Corning Life Sciences). Cancer cells were starved overnight and labeled with 5 μM Cell Tracker Red CMTPX (C34552; Thermo Fisher Scientific, Waltham, MA, USA) for 1 hour. Inserts were placed in 24-well companion plates (353504; Corning Life Sciences), coated with 150 μl of growth-reduced Matrigel matrix (356230; Corning Life Sciences), and incubated for 2 hours at 37 °C. Serum-free medium was used to seed 500 μl of starved cell suspension into the appropriate inserts and incubated at 37 °C for 24 hours. The cells that had migrated through the membrane were imaged and quantified by using a plate-based cell cytometer (Celigo; Nexcelom Bioscience LLC, Lawrence, MA, USA). Results are derived from three independent experiments with comparable outcomes (± SD).

### Aldehyde dehydrogenase activity assay

Aldehyde dehydrogenase 1 (ALDH1) activity was detected by FACS analysis using the ALDEOFLUOR assay kit (STEMCELL Technologies) according to the manufacturer‘s instructions [34]. Results are derived from three independent experiments with comparable outcomes (± SD).

### CRISPR-NOTCH3 breast cancer cells

Two custom small guide RNAs (sgRNAs) for NOTCH3 targeting were designed in silico via the CRISPR design tool (http://crispr.mit.edu:8079/). sgRNAs were cloned into an expression plasmid pSpcas9-T2A-GFP carrying sgRNA scaffold backbone, Cas9, and green fluorescent protein (GFP). Constructs were verified by sequencing and then transfected into the cells. GFP-positive cells were isolated by FACS followed by an expansion period to establish a polyclonal knockout cell population. To generate monoclonal cell lines from the polyclonal population, a limiting serial dilution protocol was used to seed individual cells in 96-well plates at an average density of 0.5 cells/well, and plates were kept in an incubator for 2 to 3 weeks. Genomic DNA was extracted from cells grown as monoclonal populations, and external primers were designed in the 5′-flanking region of sgRNAs (NOTCH3-F1: 5′-GCCAGAGGATTACCAGGAAGAGAA-3′ and Notch3-R1: 5′-CCCAGGGAAGGAGGGAGGAG-3′) were used for initial selection of knockout clones. Internal primers (NOTCH3-F1: 5′-GCCAGAGGATTACCAGGAAGAGAA-3′ and 5′-GCCAAGCTGGATTCTGTGTACCTA-3′) were used to verify prescreened clones, and the intensity of amplified product band was used as a marker for knockout efficiency. (The lower intensity is indicative of higher knockout efficiency.) Clone 416, which showed the most efficient NOTCH3 knockout, was selected and expanded, and NOTCH3 protein expression was assessed by immunoblot analysis.

### METABRIC analysis

Claudin-low breast tumor specimens were selected from participants of the METABRIC public database. The METABRIC database (http://molonc.bccrc.ca/aparicio-lab/research/metabric/) contains clinical traits, expression, copy number variation profiles, and single-nucleotide polymorphism genotypes derived from patients with breast cancer [30].

## Results

### Genomic convergence is linked to clonal expansion of breast cancer metastatic cells

**Fig. 1:**
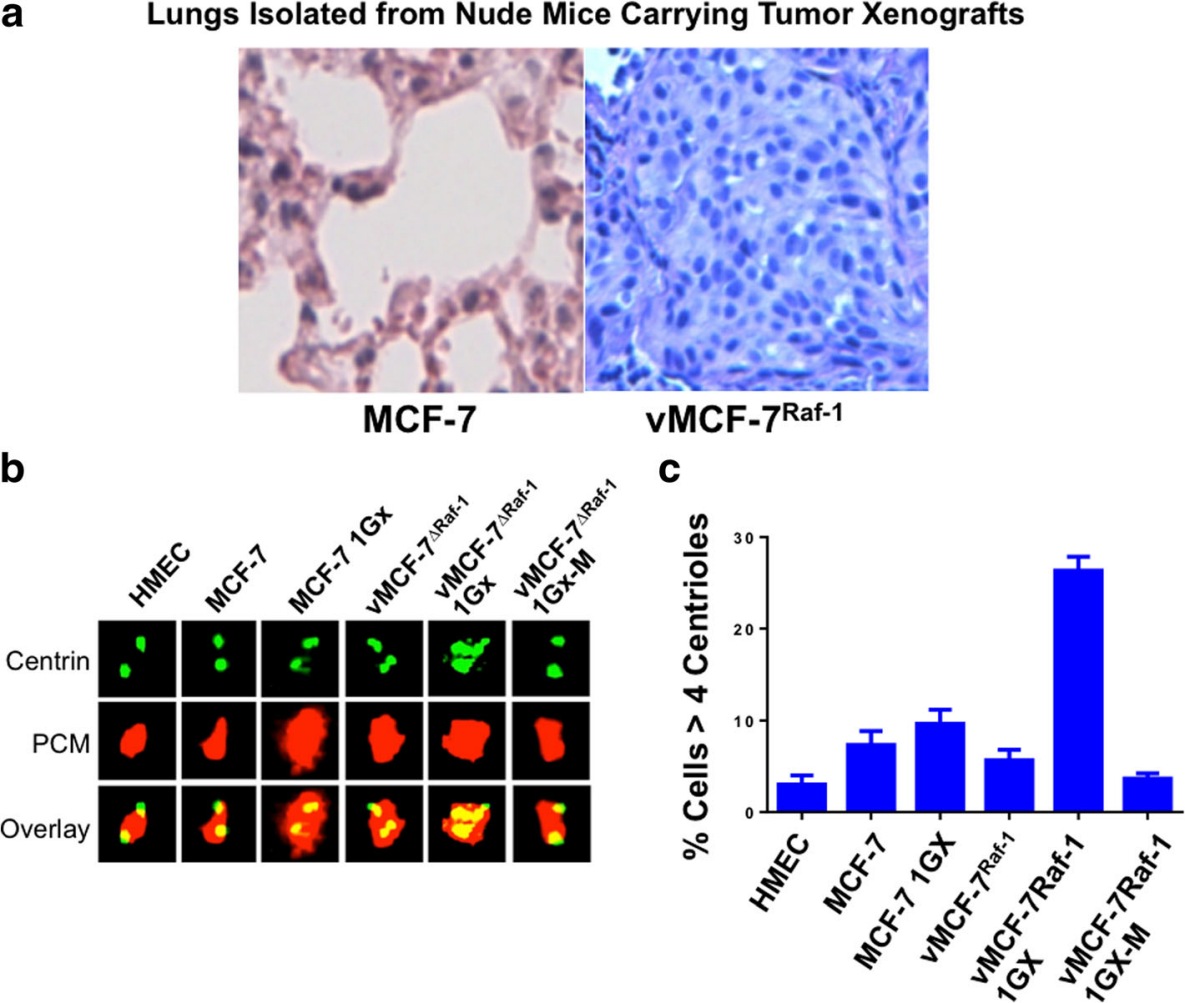
Establishment of metastatic breast cancer xenografts. **a** Lungs isolated from nude mice carrying MCF-7 and vMCF-7^∆Raf1^ tumor xenografts. Following 12 weeks of growth, animals were killed, and lung tissue was stained with H&E to determine the presence of metastatic nodules. **b** Immunofluorescence assay showing representative images of centrioles and pericentriolar material (PCM) in MCF-7 and variant breast cancer cells. Centrioles were labeled in *green* with monoclonal 20 h5 centrin antibody, and PCM was labeled in *red* with polyclonal pericentrin antibody. **c** Graph showing the average percentage of cells with more than four centrioles from three independent experiments (± SD). *HMEC* Human mammary epithelial cells

To investigate in vivo the role of Raf-1/MAPK oncogenic signaling in the genesis of BT-MICs, we used a variant MCF-7 cell line expressing a constitutive active Raf-1 oncoprotein (vMCF-7^Raf-1^) that has been described previously [5, 29]. Nude mice carrying vMCF-7^Raf-1^ xenografts were killed 12 weeks after implantation to isolate putative BT-MICs from lung metastatic nodules. Although animals carrying MCF-7 xenografts did not develop lung metastases, vMCF-7^Raf-1^ xenografts gave rise to lung micrometastatic nodules (Fig. 1a). Because breast cancer progression is functionally linked to development of centrosome amplification, which represents one of the major driving forces of chromosomal instability (CIN) [35, 36], we investigated the centrosome phenotype in MCF-7, vMCF-7^Raf-1^, and cancer cells isolated from tumor xenografts (referred to as first generation derived from xenografts [1GX]) [29]. HMEC were employed as controls because of their normal centrosome phenotype. Whereas MCF-7, MCF-7 1GX, and vMCF-7^Raf-1^ cells showed a low grade of centrosome amplification, with the majority of cells harboring two or four centrioles, centrosome amplification (more than four centrioles in a single cell) was observed in the invasive vMCF-7^Raf-1^ 1GX cells (Fig. 1b and c), in agreement with our previous findings [29]. To investigate the extent to which the degree of centrosome amplification increases during tumor progression, we also cultured metastatic cancer cells isolated from lung tissue (referred to as vMCF-7^Raf-1^ 1GX-M). Remarkably, vMCF-7^Raf-1^ 1GX-M cells showed a normal centrosome phenotype compared with their vMCF-7^Raf-1^ 1GX matching cells (Fig. 1b and c).

**Fig. 2:**
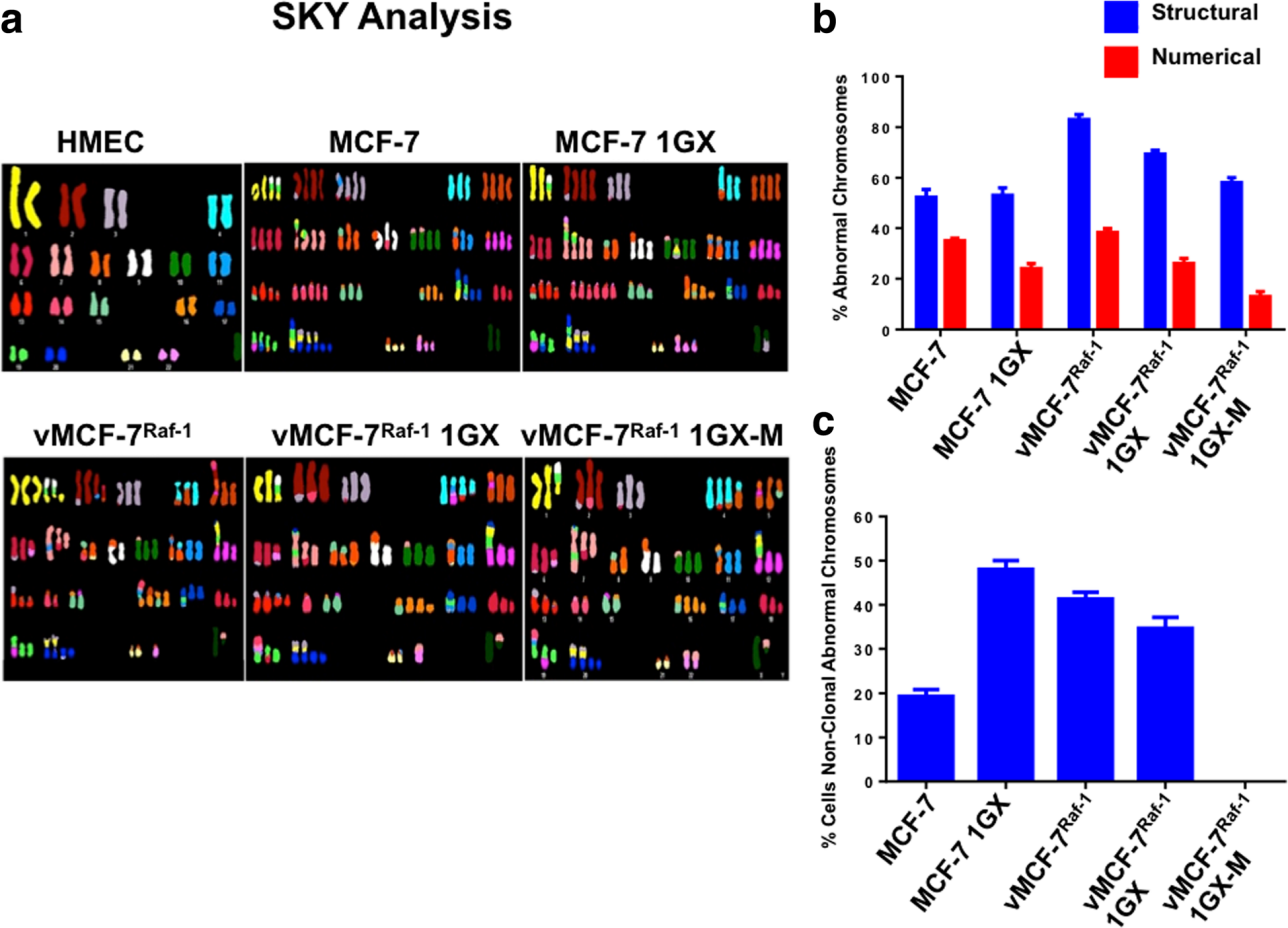
Spectral karyotyping (SKY) analysis of human breast cancer cells. **a** Representative structural and numerical chromosomal abnormalities identified through SKY analysis in MCF-7 and variant breast cancer cells. Normal human mammary epithelial cells (HMEC) were used as controls. **b** Graph showing the percentage of total structural and numerical chromosomal abnormalities identified in MCF-7 and variant breast cancer cells through SKY analysis. **c** Graph showing the percentage of nonclonal chromosomal abnormalities identified in MCF-7 and variant breast cancer cells through SKY analysis. Experiments were performed in triplicate with similar results (± SD)

To establish whether loss of centrosome amplification in vMCF-7^Raf-1^ 1GX-M cells was linked to genomic convergence and restoration of a stable karyotype, we performed SKY analysis of MCF-7 and their variant vMCF-7^Raf-1^ cells. Although MCF-7 and variant vMCF-7^Raf-1^ cells exhibited different degrees of structural and numerical chromosomal abnormalities, vMCF-7^Raf-1^ 1GX-M cells showed the lowest percentage of numerical chromosomal aberrations (Table 1 and Fig. 2a and b). In view of the fact that aneuploidy in cancer cells represents the “state,” whereas CIN indicates the “rate,” of chromosomal aberrations [36], we investigated the percentage of cells with nonclonal chromosomal abnormalities as a measure of CIN in MCF-7 and their variant vMCF-7^Raf-1^ cells. Unique chromosomal aberrations were considered nonclonal if they were present exclusively in one or two cancer cells (Table 1). Significantly, whereas nonmetastatic MCF-7 1GX cells showed the highest degree of CIN, vMCF-7^Raf-1^ 1GX-M cells exhibited only clonal chromosomal abnormalities (Table 1 and Fig. 2c).

**Table 1:**
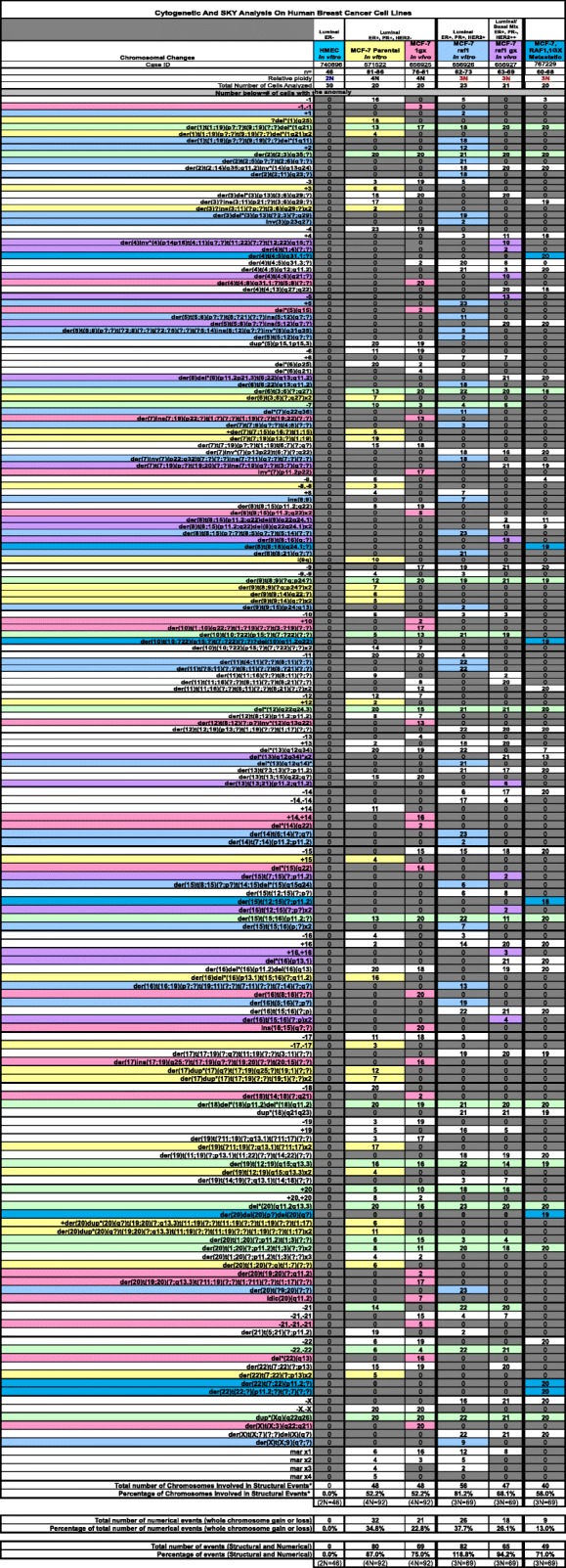
Cytogenetic and SKY Analysis of Human Mammary Epithelial Cells (HMEC) and Breast Cancer Cells: Representation of Chromosomal changes detected by cytogenetic and SKY analysis in HMEC, parental and variant MCF-7 cells

Representation of Chromosomal changes detected by cytogenetic and SKY analysis in HMEC, parental and variant MCF-7 cells

### Metastatic cells show increased self-renewal capacity that is linked to upregulation of NOTCH3 reprogramming network

**Fig. 3:**
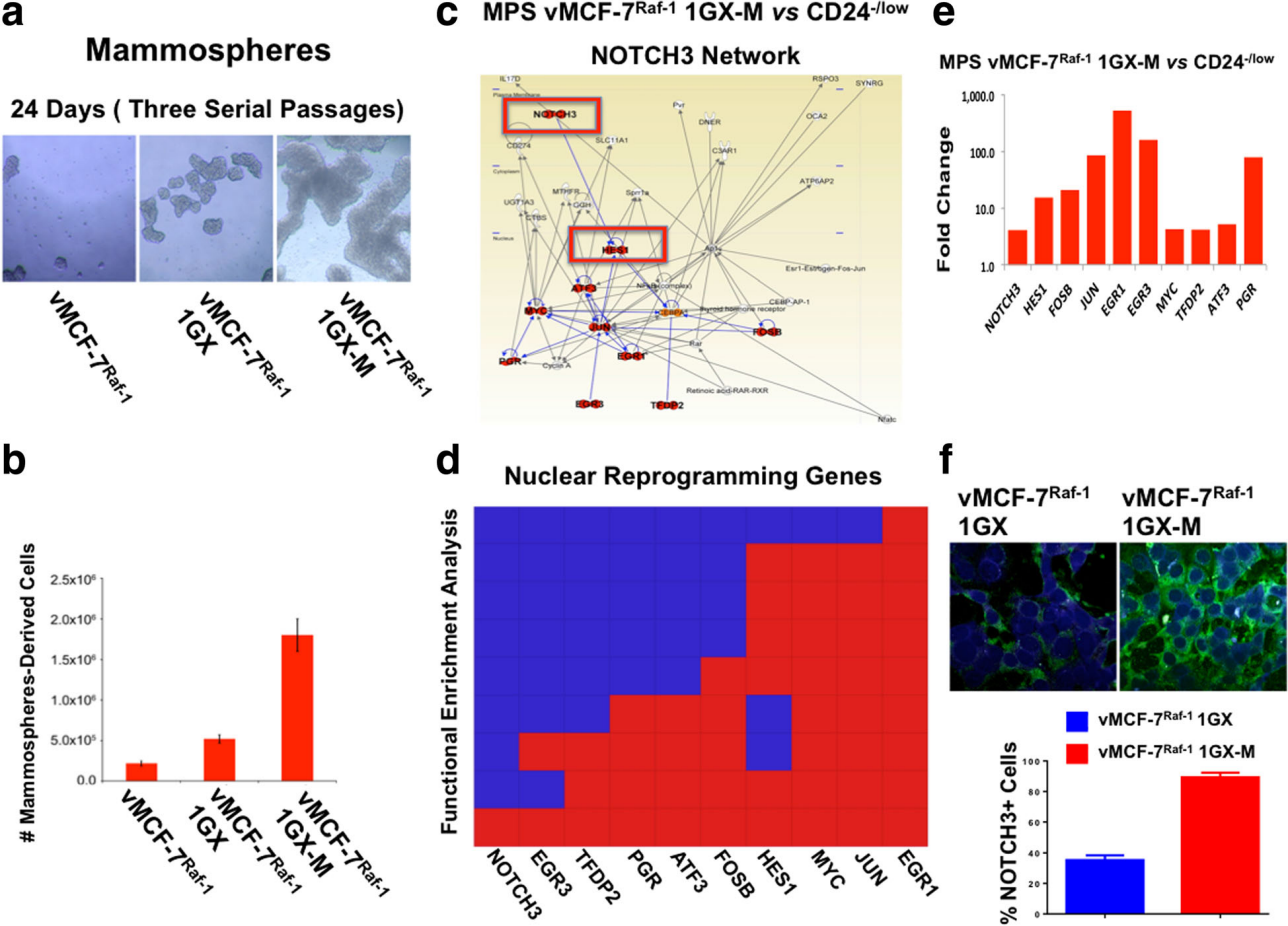
Self-renewal capacity and transcriptomic characterization of metastatic breast cancer cells. **a** Representative images of light microscopic analysis showing mammosphere (MPS) formation from vMCF-7^∆Raf1^, vMCF-7^∆Raf1^ 1GX, and vMCF-7^∆Raf1^ 1GX-M breast cancer cells after 24 days of culture under nonadherent conditions (three serial passages). **b** Graph showing the percentage of vMCF-7^∆Raf1^, vMCF-7^∆Raf1^ 1GX, and vMCF-7^∆Raf1^ 1GX-M breast cancer cells isolated from MPS after 24 days of culture under nonadherent conditions (three serial passages) from three independent experiments (± SD). **c** In Silico comparative gene network analysis between CD24^−/low^ (isolated from vMCF-7^Raf-1^ 1GX cells) and MPS vMCF-7^Raf-1^ 1GX-M cells using Ingenuity Pathway Analysis software showed upregulation of a noncanonical NOTCH3 reprogramming network that was upregulated in MPS vMCF-7^Raf-1^ 1GX-M cells. **d** In silico comparative functional enrichment analysis between CD24^−/low^ (isolated from vMCF-7^Raf-1^ 1GX cells) and MPS vMCF-7^Raf-1^ 1GX-M cells. **e** Graph showing the difference in the expression of genes identified in the NOTCH3 network between MPS vMCF-7^Raf-1^ 1GX-M and CD24^−/low^ cells. **f** Immunofluorescence analysis showing representative images of vMCF-7^∆Raf1^ 1GX and vMCF-7^∆Raf1^ 1GX-M cells stained in *green* with a NOTCH3 polyclonal antibody. Nuclei were stained in *blue* with 4′,6-diamidino-2-phenylindole. Graph showing the average of NOTCH3-expressing cells from three independent experiments (± SD)

To define whether clonal metastatic cancer cells exhibited higher stemness capacity than matching parental cells, vMCF-7^Raf-1^, vMCF-7^Raf-1^ 1GX, and vMCF-7^Raf-1^ 1GX-M cells were cultured under nonadherent conditions to test the efficiency of MPS formation that represents an excellent in vitro surrogate assay of tumor self-renewal capacity [29]. vMCF-7^Raf-1^ 1GX-M cells showed the highest number of MPS formations, demonstrating their increased ability to self-renew compared with matching parental cells (Fig. 3a and b). Because breast cancer invasiveness is functionally linked to loss or reduction of the CD24 epithelial surface marker and development of an invasive basal-like phenotype [29], we assessed CD24 expression in breast cancer cells under nonadherent conditions. Immunofluorescence analysis showed loss of CD24 expression in MPS derived from vMCF-7^Raf-1^ 1GX-M cells compared with parental cells (Additional file 1: Figure S1), demonstrating that loss of CD24 expression is linked to higher tumor self-renewal capacity and plasticity of metastatic breast cancer cells.

To identify an exclusive metastatic gene signature that typifies BT-MICs, we performed a comparative transcriptomic analysis between basal-like CD24^−/low^ (isolated by FACS from vMCF-7^Raf-1^ 1GX cells [29]) and MPS derived from vMCF-7^Raf-1^ 1GX-M (harboring a CD24^−/low^ phenotype) cells. Using a twofold change gene expression cutoff, global gene array analysis showed that 211 genes were differentially expressed between CD24^−/low^ cells and vMCF-7^Raf-1^ 1GX-M MPS (Additional file 2: Figure S2a). Functional enrichment analysis identified 59 genes involved in nuclear reprogramming that were overexpressed in vMCF-7^Raf-1^ 1GX-M MPS (Additional file 2: Figure S2b). Significantly, Ingenuity Pathway Analysis software (IPA®; QIAGEN Bioinformatics, Redwood City, CA, USA) uncovered a noncanonical NOTCH3 network that was upregulated exclusively in vMCF-7^Raf-1^ 1GX-M MPS (Fig. 3c and d). The NOTCH3 network included nine genes (HES1, FOSB, JUN, EGR1, EGR3, MYC, TFDP2, ATF3, PGR) encoding for transcription factors that play a central role in tumor progression (Fig. 3e and Additional file 3: Figure S3). Immunofluorescence analysis showed a higher percentage of vMCF-7^Raf-1^ 1GX-M cells expressing NOTCH3 than their matching vMCF-7^Raf-1^ 1GX cells (Fig. 3f), suggesting that NOTCH3-expressing cancer cells exhibited a higher capacity to promote seeding and growth to distant organs.

### NOTCH3 expression is required to induce a CD44^high^/CD24^low^/ER^low^ breast cancer stemlike phenotype, self-renewal, and invasive capacity

**Fig. 4:**
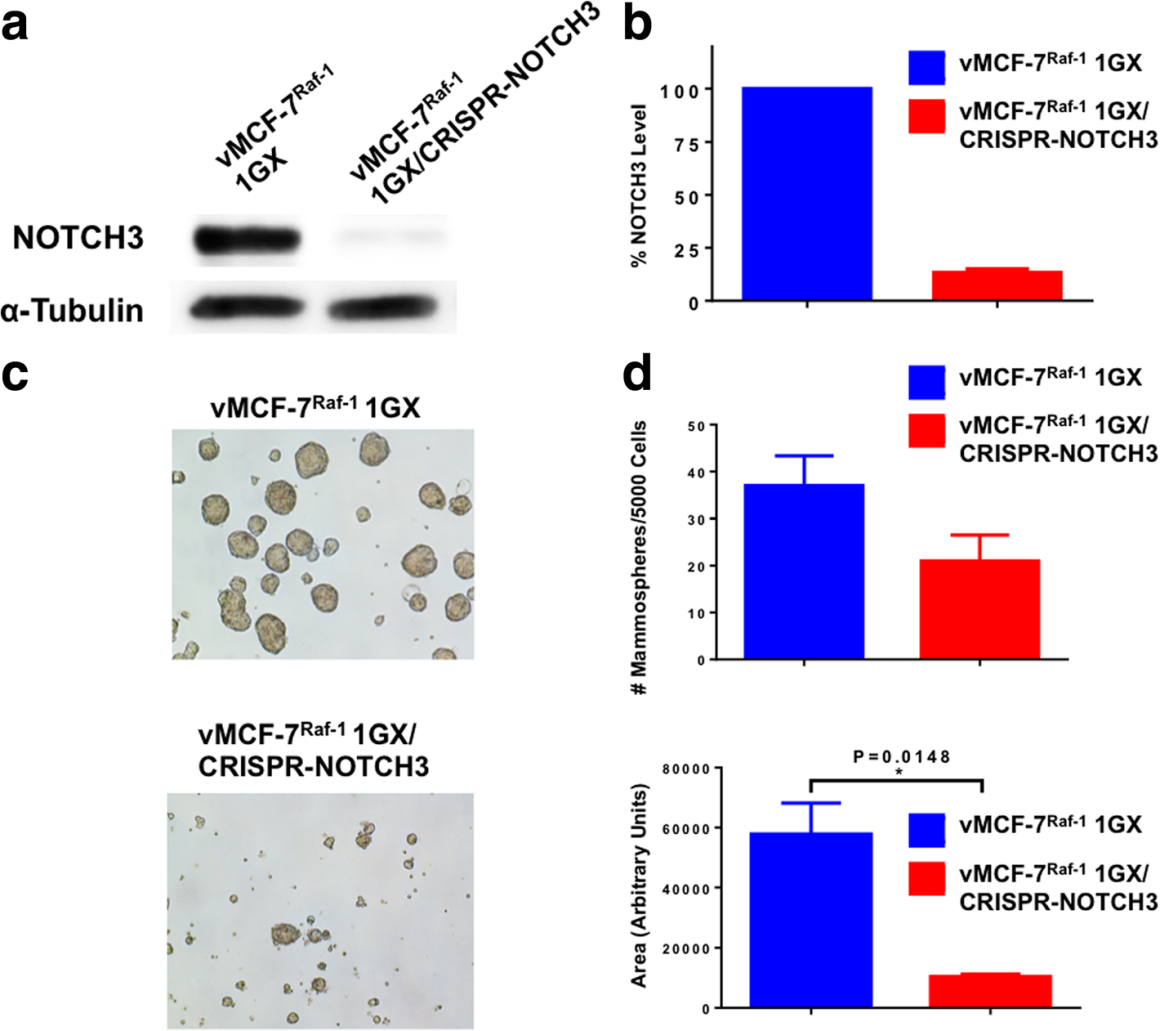
Molecular characterization of vMCF-7^∆Raf1^ 1GX cells with abrogated NOTCH3 expression. **a** Immunoblot assay showing expression of NOTCH3 in vMCF-7^∆Raf1^ 1GX and vMCF-7^∆Raf1^ 1GX/CRISPR-NOTCH3 cancer cells. **b** Densitometric analysis showing the percentage of NOTCH3 protein level in vMCF-7^∆Raf1^ 1GX/CRISPR-NOTCH3 cells relative to parental cells. Graph showing the average from three independent experiments (± SD). **c** Representative images of light microscopic analysis showing mammosphere (MPS) formation from vMCF-7^∆Raf1^ 1GX and vMCF-7^∆Raf1^ 1GX/CRISPR-NOTCH3 cells after 24 days of culture under nonadherent conditions (three serial passages). **d** Graphs showing the number and the size of MPS derived from vMCF-7^∆Raf1^ 1GX and vMCF-7^∆Raf1^ 1GX/CRISPR-NOTCH3 cells after 24 days of culture under nonadherent conditions (three serial passages). MPS size was quantified using National Institutes of Health ImageJ software (http://imagej.nih.gov/ij). Graphs show the average from three independent experiments (± SD)

**Fig. 5:**
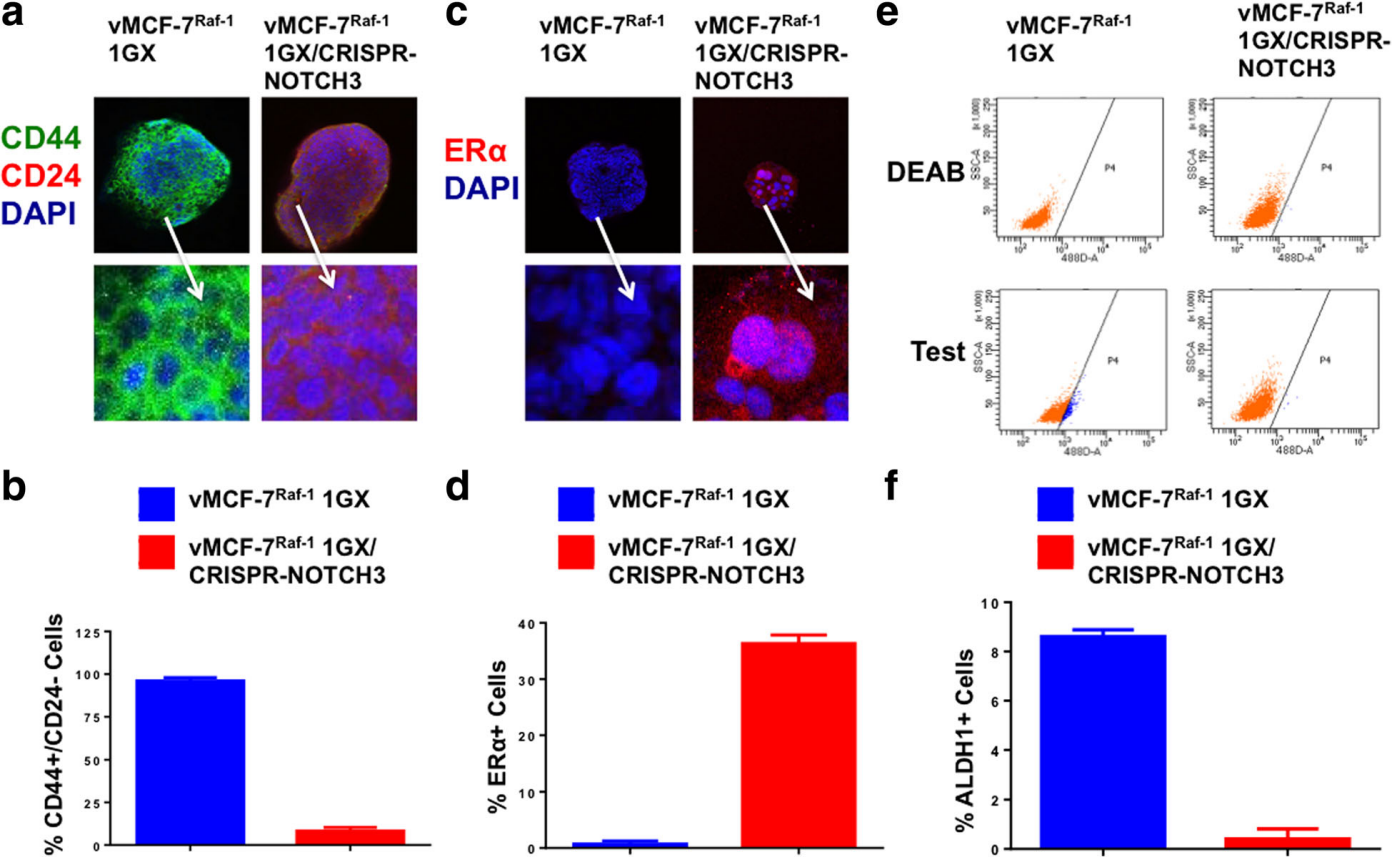
Analysis of breast cancer stemlike phenotype in vMCF-7^∆Raf1^ 1GX cells with abrogated NOTCH3 expression. **a** Immunofluorescence analysis showing representative images of vMCF-7^∆Raf1^ 1GX and vMCF-7^∆Raf1^ 1GX/CRISPR-NOTCH3 cells stained in *green* with a CD44 polyclonal antibody and in *red* with a CD24 monoclonal antibody. Nuclei were stained in *blue* with 4′,6-diamidino-2-phenylindole (DAPI). **b** Graph showing the average of cells expressing a CD44^+^/CD24^−^ phenotype from three independent experiments (± SD). **c** Immunofluorescence analysis showing representative images of vMCF-7^∆Raf1^ 1GX and vMCF-7^∆Raf1^ 1GX/CRISPR-NOTCH3 cells stained in *red* with an estrogen receptor alpha (ERα) monoclonal antibody. Nuclei were stained in *blue* with DAPI. **d** Graph showing the average of ERα-positive cells from three independent experiments (± SD). **e** Fluorescence-activated cell sorting analysis showing aldehyde dehydrogenase 1 (ALDH1) activity in vMCF-7^∆Raf1^ 1GX and vMCF-7^∆Raf1^ 1GX/CRISPR-NOTCH3 cells. Samples treated with the ALDEOFLUOR inhibitor *N,N*-diethylaminobenzaldehyde (DEAB) were used as a negative control. **f** Graph showing the average of ALDH1^+^ cells from three independent experiments (± SD)

**Fig. 6:**
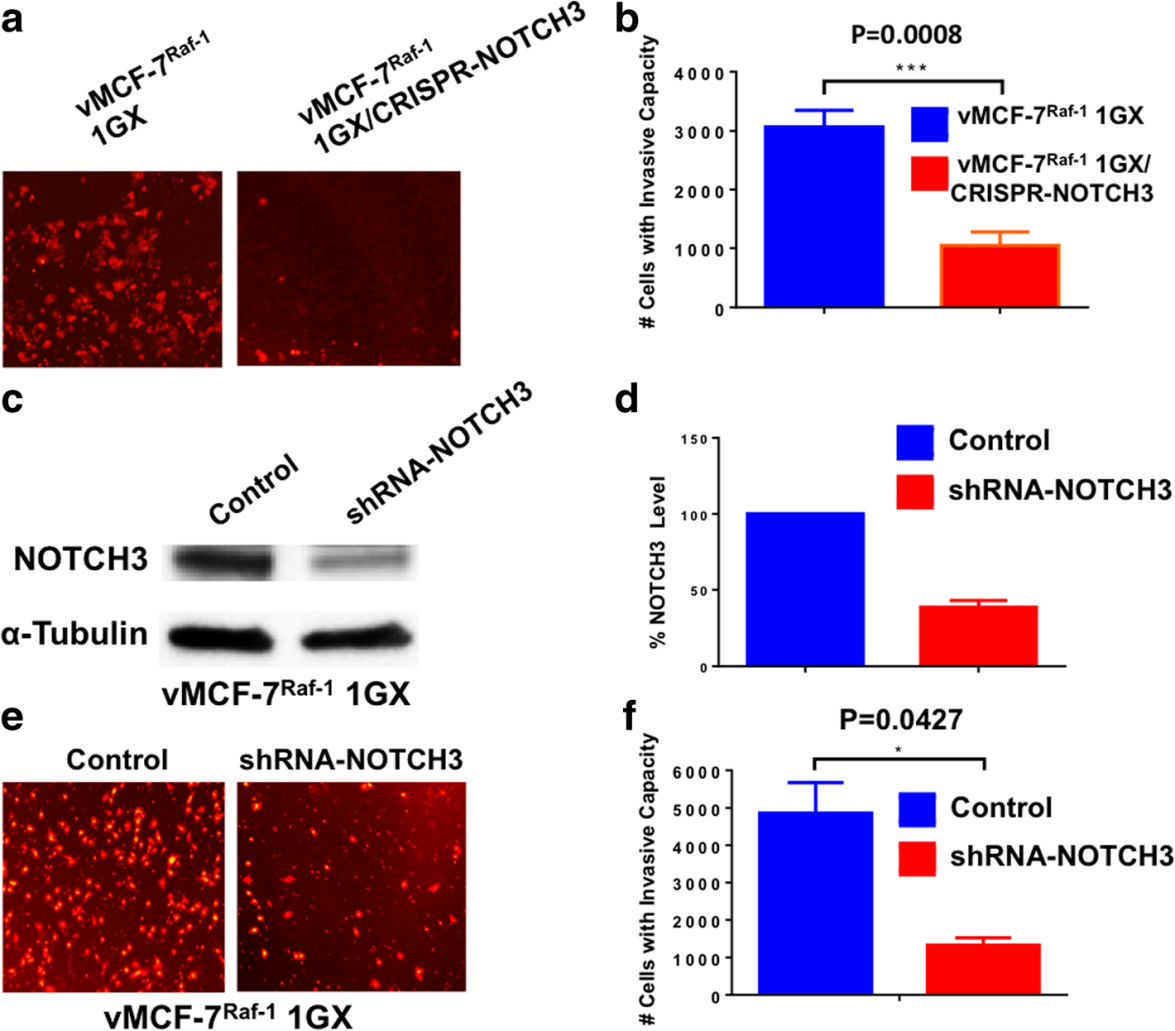
Invasive capacity of vMCF-7^∆Raf1^ 1GX cells with abrogated NOTCH3 expression. **a** In vitro real-time invasion assay of vMCF-7^∆Raf1^ 1GX and vMCF-7^∆Raf1^ 1GX/CRISPR-NOTCH3 cells stained in *red* with 5 μM Cell Tracker Red CMTPX. **b** Graph showing the average number of invasive cells from three independent experiments (± SD). **c** Immunoblot assay showing NOTCH3 expression in vMCF-7^∆Raf1^ 1GX cells infected with scramble lentivirus short hairpin RNA (lenti-shRNA; control) and lenti-shRNAs targeting NOTCH3 messenger RNA (mRNA). **d** Densitometric analysis showing the percentage of NOTCH3 protein level in vMCF-7^∆Raf1^ 1GX/shRNA-NOTCH3 cells relative to control. Graph showing the average from three independent experiments (± SD). **e** In vitro real-time invasion assay of vMCF-7^∆Raf1^ 1GX cells infected with scramble lenti-shRNAs (control) and lenti-shRNAs targeting NOTCH3 mRNA. **f** Graph showing the average number of invasive cells from three independent experiments (± SD)

Because our results indicate that NOTCH3-expressing cells originate in vMCF-7^∆Raf1^ xenografts (Fig. 3f), we employed the CRISPR-Cas9 gene editing technology to generate unique NOTCH3-knockout breast cancer cells (vMCF-7^Raf-1^ 1GX^CRISPR-NOTCH3^) and assessed their stemness and invasive properties (Additional file 4: Figure S4 and Fig. 4a and b). vMCF-7^Raf-1^ 1GX and vMCF-7^Raf-1^ 1GX^CRISPR-NOTCH3^ cells were cultured under nonadherent conditions to test the efficiency of MPS formation. vMCF-7^Raf-1^ 1GX^CRISPR-NOTCH3^ cells exhibited a reduction in the number and size of MPS formation compared with parental vMCF-7^Raf-1^ 1GX cells (Fig. 4c and d). To define the extent to which impairment of self-renewal capacity was linked to suppression of breast cancer stemlike phenotype, vMCF-7^Raf-1^ 1GX and vMCF-7^Raf-1^ 1GX^CRISPR-NOTCH3^ MPS were stained for CD44 and CD24 breast cancer stemness markers. vMCF-7^Raf-1^ 1GX^CRISPR-NOTCH3^ MPS exhibited a more differentiated CD44^low^/CD24^high^ phenotype compared with vMCF-7^Raf-1^ 1GX MPS that showed a CD44^high^/CD24^low^ cancer stemlike phenotype (Fig. 5a and b). Because CD44^high^/CD24^low^ breast cancer stemlike cells also lack ERα expression [29, 37], we aimed to assess ERα expression/localization in vMCF-7^Raf-1^ 1GX and vMCF-7^Raf-1^ 1GX^CRISPR-NOTCH3^ MPS. Whereas vMCF-7^Raf-1^ 1GX MPS lacked nuclear ERα expression, partial restoration of nuclear ERα expression was observed in vMCF-7^Raf-1^ 1GX^CRISPR-NOTCH3^ MPS (Fig. 5a and d), corroborating the role of the NOTCH3 signaling pathway in restraining ERα expression in breast cancer cells [38]. To define the causative role of NOTCH3 expression in promoting ALDH1 activity that represents a universal functional marker of tumor stemness, chemoresistance, and metastasis [34, 39], we performed an ALDEOFLUOR assay in vMCF-7^Raf-1^ 1GX and vMCF-7^Raf-1^ 1GX^CRISPR-NOTCH3^ cells. Significantly, vMCF-7^Raf-1^ 1GX^CRISPR-NOTCH3^ cells showed a reduction of ALDH1 activity compared with parental vMCF-7^Raf-1^ 1GX cells (Fig. 5e and f). Last, we aimed to investigate whether lack of NOTCH3 expression was linked to an impairment of vMCF-7^Raf-1^ 1GX cells’ invasive capacity. An in vitro real-time invasion assay showed that abrogation of NOTCH3 expression significantly reduced the invasiveness of vMCF-7^Raf-1^ 1GX cells (Fig. 6a and b). These results were validated in vMCF-7^Raf-1^ 1GX cells infected with lentiviral short hairpin RNAs (lenti-shRNAs) targeting NOTCH3 messenger RNAs (mRNAs) that also showed a significant impairment of invasive capacity (Fig. 6c–f).

### NOTCH3 expression is necessary to mediate AURKA-induced invasiveness of breast cancer cells

**Fig. 7:**
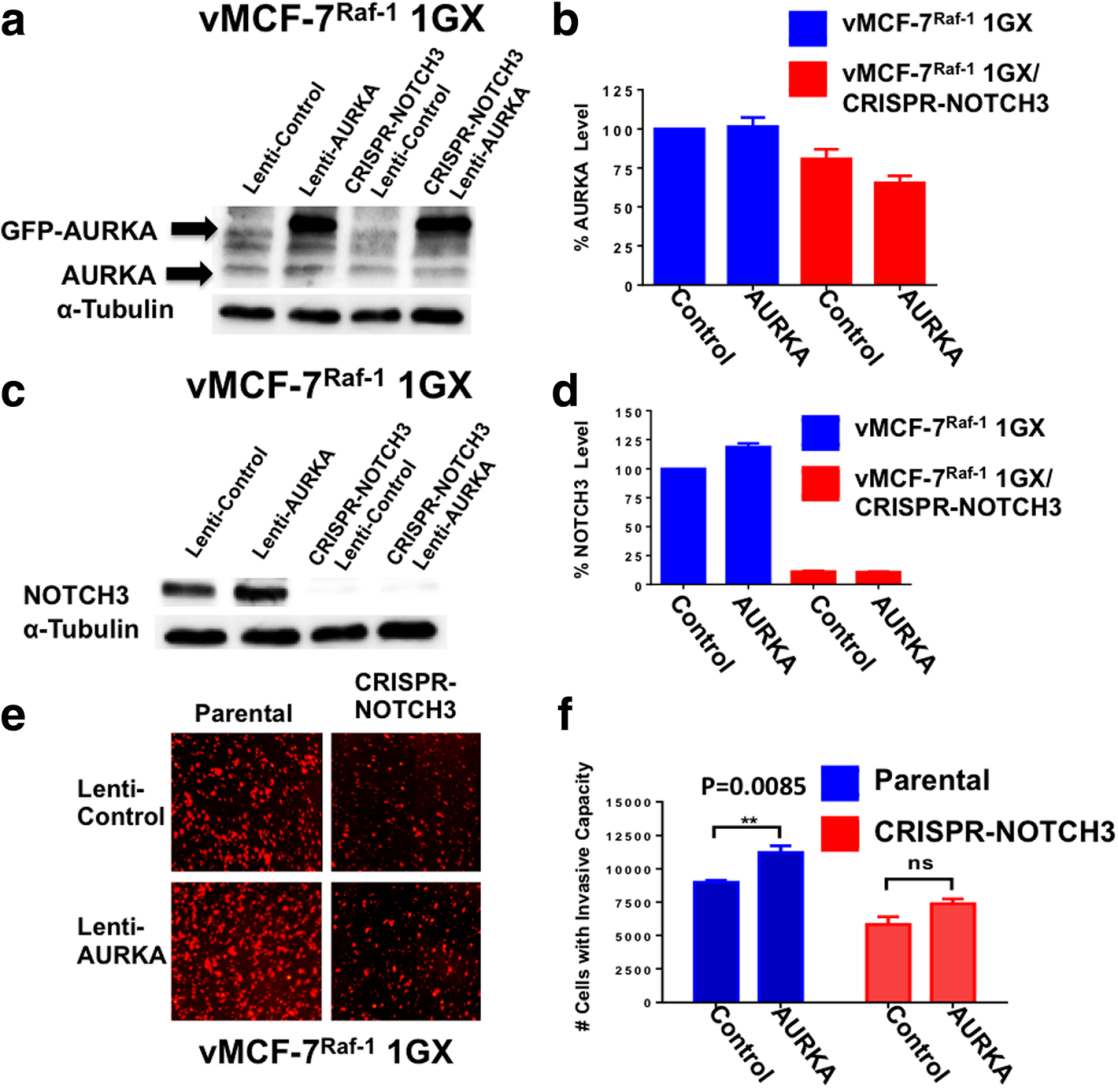
Invasive capacity of vMCF-7^∆Raf1^ 1GX cells expressing a green fluorescent protein (GFP)-tagged kinase Aurora kinase A (AURKA) construct. **a** Immunoblot assay showing expression of endogenous and GFP-tagged AURKA in vMCF-7^∆Raf1^ 1GX and vMCF-7^∆Raf1^ 1GX/CRISPR-NOTCH3 cells. **b** Densitometric analysis showing the percentage of endogenous AURKA protein levels in vMCF-7^∆Raf1^ 1GX and vMCF-7^∆Raf1^ 1GX/CRISPR-NOTCH3 cells relative to control. Graph shows the average from three independent experiments (± SD). **c** Immunoblot assay showing NOTCH3 protein levels in vMCF-7^∆Raf1^ 1GX and vMCF-7^∆Raf1^ 1GX/CRISPR-NOTCH3 cells expressing empty lentiviral vectors (control) and lentiviral GFP-tagged AURKA vectors. **d** Densitometric analysis showing the percentage of NOTCH3 protein levels in vMCF-7^∆Raf1^ 1GX and vMCF-7^∆Raf1^ 1GX/shRNA-NOTCH3 cells relative to vMCF-7^∆Raf1^ 1GX cells infected with empty lentiviral vectors (control). Graph shows the average from three independent experiments (± SD). **e** In vitro real-time invasion assay of vMCF-7^∆Raf1^ 1GX and vMCF-7^∆Raf1^ 1GX/CRISPR-NOTCH3 cells expressing empty lentiviral vectors (control) and lentiviral GFP-tagged AURKA vectors. **f** Graph showing the average number of invasive cells from three independent experiments (± SD)

Because we have previously demonstrated in vMCF-7^∆Raf1^ xenografts the causal role of aberrant AURKA activity in inducing the development of spontaneous lung metastases [29], we investigated whether NOTCH3 expression was necessary to mediate AURKA-induced invasive capacity of vMCF-7^∆Raf1^ 1GX cells. vMCF-7^∆Raf1^ 1GX and vMCF-7^Raf-1^ 1GX^CRISPR-NOTCH3^ cells were infected with empty lentiviral vectors (lenti-vectors; used as control) and lenti-vectors expressing a GFP-tagged AURKA construct (Fig. 7a and b). Endogenous levels of AURKA were reduced in vMCF-7^Raf-1^ 1GX^CRISPR-NOTCH3^ cells, whereas only vMCF-7^∆Raf1^ 1GX cells expressing GFP-AURKA showed increased NOTCH3 expression (Fig. 7a–d). These results demonstrate a positive feedback loop between AURKA and NOTCH3 oncogenic pathways. To define whether NOTCH3 expression was essential to mediate AURKA-induced invasiveness of vMCF-7^∆Raf1^ 1GX cells, we performed an in vitro real-time invasion assay. Significantly, expression of GFP-AURKA in vMCF-7^∆Raf1^ 1GX cells enhanced their invasive capacity (Fig. 7e and f). On the contrary, expression of GFP-AURKA in vMCF-7^Raf-1^ 1GX^CRISPR-NOTCH3^ cells failed to restore their invasive ability (Fig. 7e and f), demonstrating that NOTCH3 expression is required to mediate AURKA-induced breast cancer cells’ aggressiveness.

### Pharmacologic targeting of NOTCH signaling inhibits TNBC cell seeding and metastatic growth

**Fig. 8:**
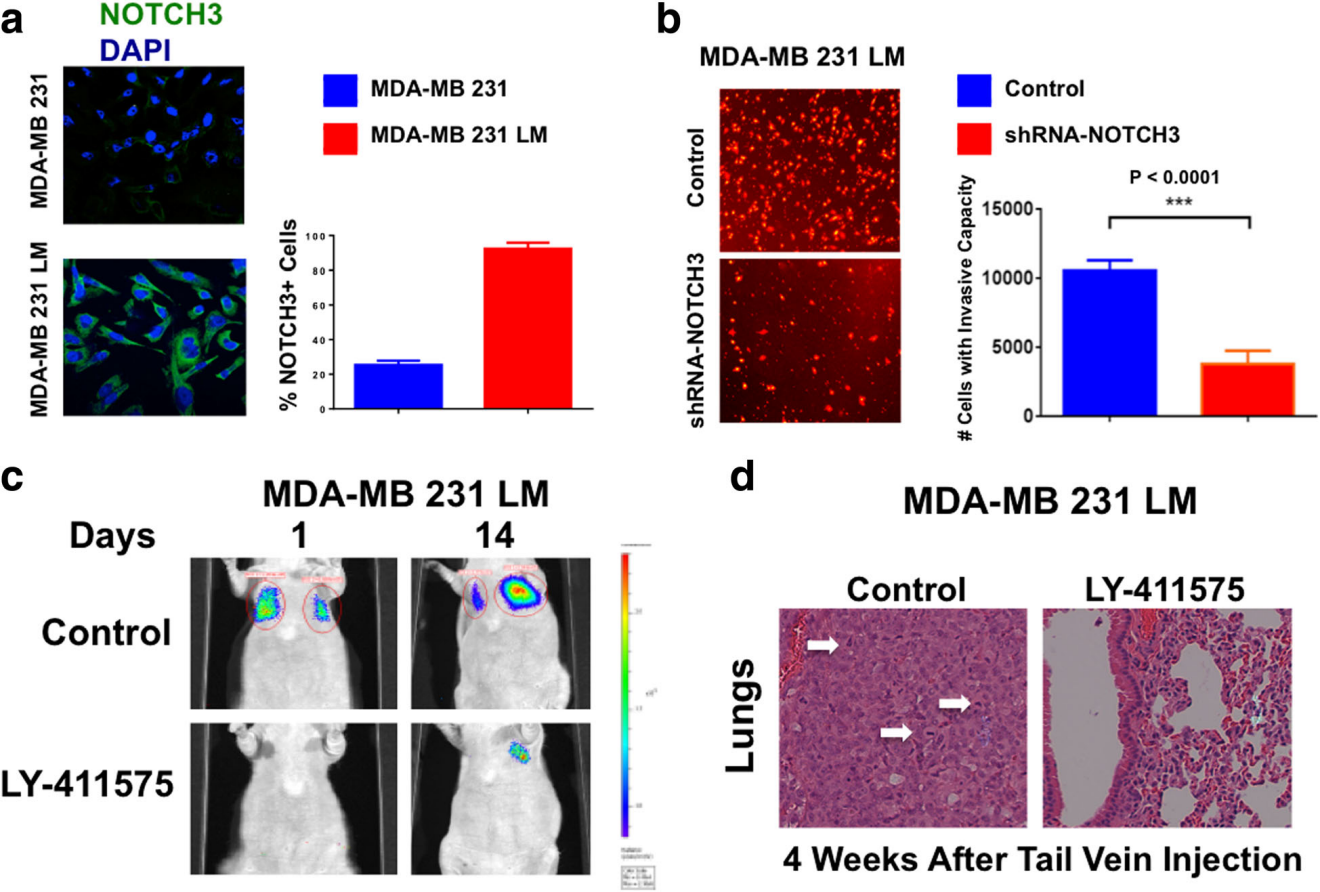
Pharmacologic targeting of NOTCH signaling in triple-negative breast cancer (TNBC) cells. **a** Immunofluorescence analysis showing representative images of MDA-MB-231 and MDA-MB-231 lung metastasis (LM) TNBC cells stained in *green* with a NOTCH3 polyclonal antibody. Nuclei are stained in *blue* with 4′,6-diamidino-2-phenylindole (DAPI). Graph shows the average number of NOTCH3-expressing cells from three independent experiments (± SD). **b** In vitro real-time invasion assay of MDA-MB-231 LM TNBC cells infected with scramble lentivirus short hairpin RNAs (lenti-shRNAs; control) and lenti-shRNAs targeting NOTCH3 messenger RNA. Graph shows the average number of invasive cells from three independent experiments (± SD). **c** Experimental lung metastasis imaging in live animals of LY-411575-treated or dimethyl sulfoxide (DMSO)-treated MDA-MB-231 LM cells expressing the firefly luciferase reporter lentivector after tail vein injection. **d** Lungs isolated from nude mice that were injected with LY-411575-treated or DMSO-treated MDA-MB-231 LM TNBC cells. Following 4 weeks of growth, animals were killed, and lung tissues were stained with H&E to determine the presence of metastatic lesions as previously described [29]

To confirm in a different breast cancer model the finding that NOTCH3 expression is restricted to metastatic cells, we used CD44^high^/CD24^low^ MDA-MB-231 TNBC cells isolated from experimental lung metastases (MDA-MB-231 LM) [37, 40]. MDA-MB-231 LM cells showed a higher percentage of cells expressing NOTCH3 than parental MDA-MB-231 cells (Fig. 8a). To assess the causal role of NOTCH3 expression in promoting the highly invasive capacity of MDA-MB-231 LM cells, we employed an in vitro real-time invasion assay. MDA-MB-231 LM cells infected with lenti-shRNAs targeting NOTCH3 significantly reduced their invasiveness compared with MDA-MB-231 LM cells infected with scramble lenti-shRNAs used as control (Fig. 8b). Next, we aimed to establish whether pharmacologic targeting of NOTCH signaling inhibits metastatic seeding and growth of cancer cells overexpressing NOTCH3. MDA-MB-231 LM cells were treated in vitro with 500 nM of LY411575 (pan-NOTCH inhibitor), and MDA-MB-231 LM cells treated with vehicle dimethyl sulfoxide (DMSO) were used as a control. After 48-hour incubation, treated and control MDA-MB-231 LM cells were washed with PBS and cultured in drug-free medium for an additional 48 hours. Viable cells were then injected into the tail vein of immune-compromised mice to develop experimental lung metastases as previously described [29]. Whereas animals injected with DMSO-treated MDA-MB-231 LM cells developed lung metastases, animals injected with LY411575-treated MDA-MB-231 LM cells showed impaired lung metastatic lesions (Fig. 8c). Histopathologic analysis confirmed that whereas lungs isolated from control animals exhibited metastatic lesions with several mitotic figures indicative of active proliferating cancer cells, the alveolar structure of lungs isolated from LY411575-treated animals was largely preserved (Fig. 8d). Importantly, because MDA-MB-231 LM and parental cells showed nominal levels of NOTCH1 and NOTCH2 (Additional file 5: Figure S5a and b), these results suggest that LY411575-mediated inhibition of cancer cell seeding and metastatic growth was likely associated with NOTCH3 targeting.

### Genetic targeting of NOTCH3 reduces the self-renewal and invasive capacity of patient-derived brain metastasis TNBC cells

**Fig. 9:**
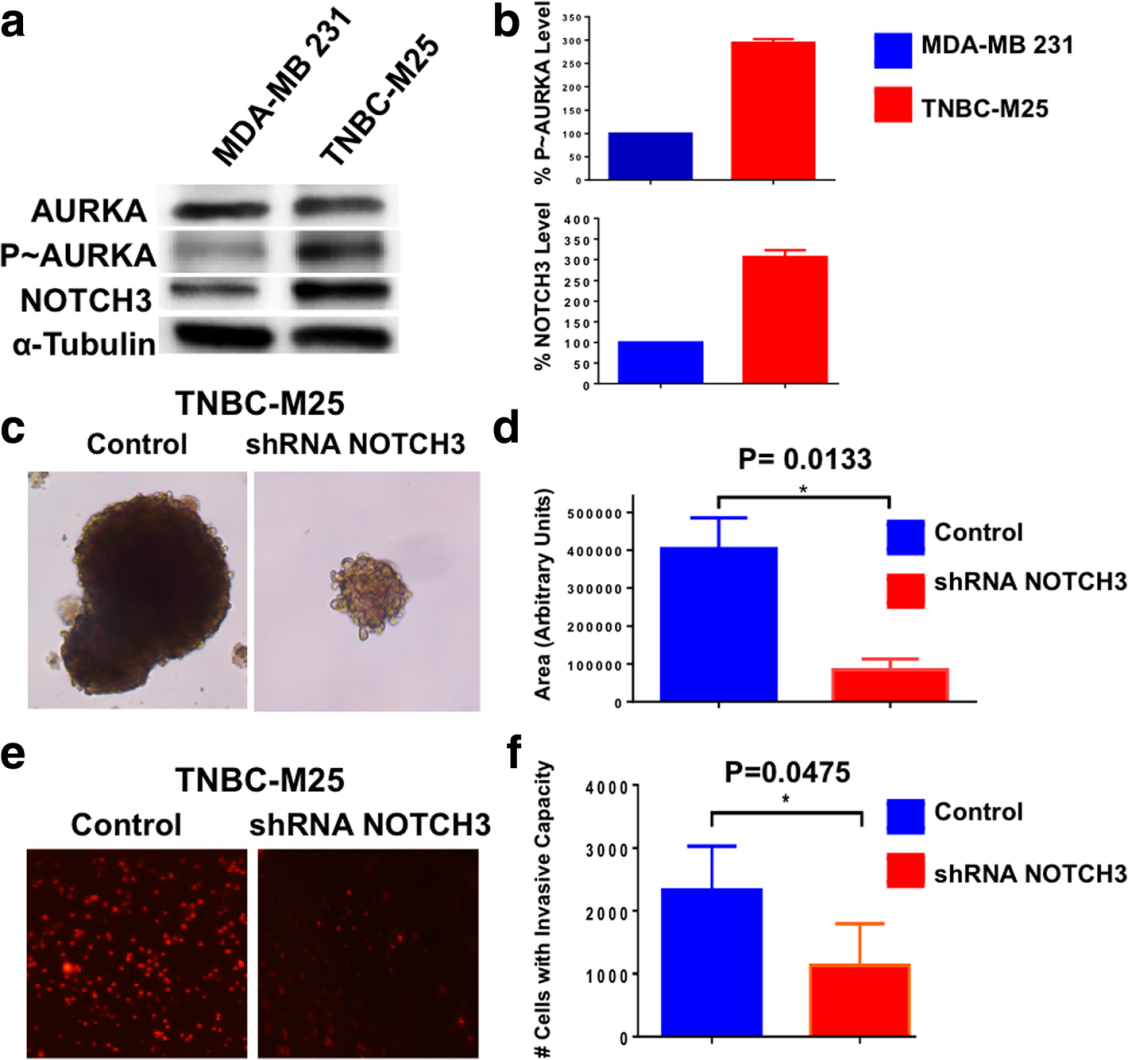
Self-renewal and invasive capacity of patient-derived triple-negative breast cancer (TNBC) cells. **a** Immunoblot assay showing total Aurora kinase A (AURKA), phosphorylated AURKA (p~AURKA), and NOTCH3 expression in MDA-MB-231 and patient-derived TNBC-M25 cells. **b** Densitometry analysis showing the percentage of p~AURKA and NOTCH3 protein levels in TNBC-M25 cells relative to MDA-MB-231 cells. Graph shows the average from three independent experiments (± SD). **c** Representative images of light microscopic analysis showing single-cell dilution tertiary mammosphere (MPS) from TNBC-M25 cells infected with scramble lentiviral short hairpin RNAs (lenti-shRNAs; control) and lenti-shRNAs targeting NOTCH3 messenger RNA (mRNA). **d** Graphs showing the average size from three independent experiments (± SD) of tertiary MPS derived from TNBC-M25 cells infected with scramble lenti-shRNAs (control) and lenti-shRNAs targeting NOTCH3 mRNA. MPS size was quantified using the National Institutes of Health ImageJ software (http://imagej.nih.gov/ij). **e** In vitro real-time invasion assay of TNBC-M25 cells infected with scramble lenti-shRNAs (control) and lenti-shRNAs targeting NOTCH3 mRNA. **f** Graph showing the average number of invasive cells from three independent experiments (± SD)

To validate in primary breast cancer cells the central role of the NOTCH3 signaling pathway in inducing a metastatic phenotype, we developed unique TNBC cells (TNBC-M25) isolated from a patient-derived brain metastasis xenograft model that was generated by the BEAUTY trial in the Mayo Clinic [32]. TNBC-M25 cells showed increased expression of phospho-AURKA and NOTCH3 compared with MDA-MB-231 cells (Fig. 9a and b). Significantly, TNBC-M25 cells showed low levels of NOTCH1 and NOTCH2, suggesting that NOTCH3 expression plays a major role in promoting their metastatic phenotype (Additional file 6: Figure S6a and b). To define the causative role of NOTCH3 expression in inducing self-renewal capacity, TNBC-M25 cells were infected with lenti-shRNAs targeting NOTCH3 (TNBC-M25 infected with scramble lenti-shRNAs were used as a control) and were cultured under nonadherent conditions to test the efficiency of MPS formation. TNBC-M25 cells infected with lenti-shRNA NOTCH3 exhibited a significant reduction in the size of MPS formation compared with control cells (Fig. 9c and d). To investigate the extent to which impairment of self-renewal ability was linked to inhibition of invasiveness in TNBC-M25 cells with reduced NOTCH3 expression, we employed an in vitro real-time invasion assay. TNBC-M25 cells infected with lenti-shRNA NOTCH3 significantly reduced their invasive capacity compared with TNBC-M25 cells infected with scramble lenti-shRNAs used as a control (Fig. 9c and f).

### Aberrant NOTCH3 expression is linked to shorter overall survival of patients with breast cancer

**Fig. 10:**
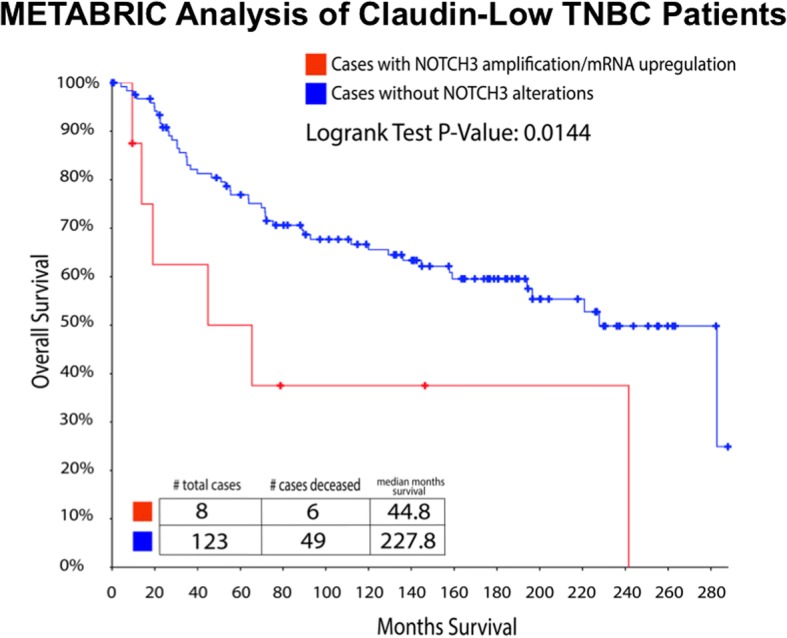
Molecular Taxonomy of Breast Cancer International Consortium (METABRIC) analysis of claudin-low triple-negative breast cancer (TNBC) patients. The claudin-low subgroup analyzed in the METABRIC study represented a cluster of 125 patients characterized by 112 TNBC and 13 ER^−^/PR^−^/HER2^+^ specimens, and the average of age at diagnosis was 56.9 years. NOTCH3 alterations characterized by messenger RNA upregulation and/or copy number variations were detected in 8 of 117 cases (6 TNBC and 2 ER^−^/PR^−^/HER2^+^). Cases of death involved 6 patients with aberrant NOTCH3 expression and 46 patients without NOTCH3 alterations. Survival analysis showed that NOTCH3 expression was significantly associated with decreased overall survival (*p* = 0.0145)

We analyzed the genome sequencing and mRNA-sequencing data of specimens from the METABRIC study [30] to define the linkage between NOTCH3 expression and overall survival of patients with claudin-low breast tumors. The claudin-low subgroup analyzed in the METABRIC study represented a cluster of 125 patients characterized by 112 TNBC and 13 ER^−^/PR^−^/HER2^+^ specimens, and the average age at diagnosis was 56.9 years. Whereas none of the 125 patients harbored any deletion or downregulation of NOTCH3, 8 of 117 cases (6 TNBC and 2 ER^−^/PR^−^/HER2^+^) showed NOTCH3 alterations characterized by mRNA upregulation and/or copy number variations. Cases of death involved 6 patients with aberrant NOTCH3 expression and 46 patients without NOTCH3 alterations. Our survival analysis showed that NOTCH3 expression was significantly associated with decreased overall survival (*p* = 0.0145). Specifically, the average of the overall survival (calculated for the whole follow-up) was 282.8 months for the cases without NOTCH3 alterations and 44.8 months for the cases with aberrant NOTCH3 expression (Fig. 10).

## Discussion

Development of distant metastases involves a complex multistep biological process termed the *invasion-metastasis cascade*, which includes dissemination of cancer cells from the primary tumor to secondary organs [41]. This process is inefficient because it has been estimated that less than 1% of cancer cells will be successful in establishing clinically detectable metastatic lesions [42]. Specifically in breast cancer, BT-MICs must go through EMT, invade the extracellular matrix, intravasate and survive in the systemic circulation, extravasate at the metastatic site, and finally seed in the new microenvironment [42–44]. Importantly, each of these events is driven by the accumulation of genetic and/or epigenetic alterations within cancer cells necessary for the clonal selection and expansion of BT-MICs that ultimately give rise to distant metastases [44]. Several lines of evidence support the hypothesis that BT-MICs might be found within subpopulations of BTICs [46]. In support of this hypothesis, it has been shown that oncogenic pathways such as MAPK, AURKA, and NOTCH that induce EMT and expansion of BTICs also promote onset of distant metastases [5, 29, 47, 48]. The characterization of the precise role of each of these oncogenic pathways in the sequential stepwise events that typify the invasion-metastasis cascade will be essential for the development of precise therapeutic strategies aimed at eradicating distant metastases.

In this study, we uncovered the linkage between NOTCH3 expression and development of distant metastases in experimental breast cancer models. First, we used luminal ER^+^ MCF-7 and variant cells with constitutively active Raf-1/MAPK signaling (vMCF-7^∆Raf1^) to establish in vivo the association between aberrant Raf-1/MAPK signaling, CIN, and onset of distant metastases. In agreement with our previous studies [5, 29], only vMCF-7^∆Raf1^ tumor xenografts developed spontaneous lung metastases, corroborating the causal role of aberrant activation of Raf-1/MAPK pathway in promoting metastatic lesions. Significantly, ex vivo cancer cells isolated from lung metastases (vMCF-7^∆Raf1^ 1GX-M) showed a normal centrosome phenotype and clonal chromosomal aberrations compared with matching vMCF-7^∆Raf1^ 1GX parental cells that exhibited centrosome amplification and nonclonal chromosomal aberrations resulting in CIN. These findings demonstrate in vivo that loss of centrosome amplification is linked to restoration of chromosomal stability resulting in the clonal expansion of metastatic cancer cells. Moreover, they support the genomic convergence model proposed for tumor progression in which CIN initially imposed during tumorigenesis becomes suppressed when cancer cells have acquired the suitable chromosome compositions and gene dosage that will lead to the successful establishment of distant metastases [48]. Because it has been hypothesized that BT-MICs are late stages BTIC subclones with higher stemness capacity [45], we cultured vMCF-7^∆Raf1^ 1GX-M and matching vMCF-7^∆Raf1^ and vMCF-7^∆Raf1^ 1GX parental cells under nonadherent conditions to form MPS as an in vitro surrogate assay of self-renewal capacity. vMCF-7^∆Raf1^ 1GX-M cells showed the highest efficiency in MPS formation compared with matching parental cells. This increased self-renewal capacity was linked to loss of the CD24 epithelial marker in vMCF-7^∆Raf1^ 1GX-M MPS. These results demonstrate that chromosomal stable vMCF-7^∆Raf1^ 1GX-M cells have acquired higher self-renewal and CD24^−/low^ basal-like plasticity that plays a critical role in EMT, cancer cell seeding, and metastatic growth to secondary organs [29]. Next, we wanted to establish whether chromosomal stability and high self-renewal capacity of vMCF-7^∆Raf1^ 1GX-M cells was linked to an exclusive metastatic signature. To answer this question, we performed unbiased comparative transcriptomic and functional gene enrichment analyses between MPS vMCF-7^∆Raf1^ 1GX-M (that show CD24^−/low^) and highly invasive CD24^−/low^ basal-like cells isolated from matching vMCF-7^∆Raf1^ tumor xenografts as previously demonstrated [29]. Functional gene enrichment analysis identified a noncanonical NOTCH3 reprogramming network that was upregulated in vMCF-7^∆Raf1^ 1GX-M MPS. The NOTCH3 network comprised nine genes encoding for transcriptional factors with high oncogenic activity (HES1, FOSB, JUN, EGR1, EGR3, MYC, TFDP2, ATF3, PGR). This reprogramming network included the NOTCH downstream target HES1, suggesting that NOTCH3/HES1 stemness signaling may play a central role in promoting the survival and seeding of BT-MICs to secondary organs. Because detection of cancer cell seeding to secondary organs and onset of micrometastases is clinically challenging, expression of the NOTCH3 metastatic signature in circulating tumor cells may have promising clinical relevance in predicting early onset of distant metastases in patients with breast cancer. Importantly, the majority of vMCF-7^∆Raf1^ 1GX-M cells were strongly positive for NOTCH3 staining by immunofluorescence assay, demonstrating that NOTCH3 expression was restricted to clonal metastatic breast cancer cells. vMCF-7^∆Raf1^ 1GX xenografts exhibiting CIN showed tumor cell heterogeneity for NOTCH3 expression, indicating that NOTCH3^high^-expressing subclones may arise from the primary tumor and promote distant metastasis owing to their higher stemness capacity, in agreement with the recent finding that metastatic clones disseminate early from primary breast tumors [49]. On the basis of these results, we developed unique NOTCH3-knockout breast cancer cells (vMCF-7^Raf-1^ 1GX^CRISPR-NOTCH3^) to abrogate NOTCH3 expression and evaluate the causative role of NOTCH3 signaling in promoting stemness and invasive properties of vMCF-7^∆Raf1^ 1GX cells. NOTCH3 expression was required to induce in vitro self-renewal capacity and a CD44^high^/CD24^low^ breast cancer stemlike phenotype in vMCF-7^∆Raf1^ 1GX cells. Because we and others have demonstrated that CD44^high^/CD24^low^ BTICs also show an ERα^low/−^ basal-like phenotype [29, 37, 38], we assessed ERα in MPS derived from vMCF-7^Raf-1^ 1GX^CRISPR-NOTCH3^ and parental cells. Whereas MPS derived from vMCF-7^Raf-1^ 1GX cells lacked ERα expression, MPS resulting from vMCF-7^Raf-1^ 1GX^CRISPR-NOTCH3^ cells exhibited restoration of ERα expression, compatible with previous studies that demonstrated the role of NOTCH3 signaling in suppressing ERα expression [38]. Because high ALDH1 activity has been linked to stemness, early onset of distant metastases, and poor prognosis in breast cancer [39], we performed an ALDEOFLUOR assay that accurately identifies highly tumorigenic cancer cells with elevated ALDH1 activity. Importantly, vMCF-7^Raf-1^ 1GX^CRISPR-NOTCH3^ cells showed minimal ALDH1 activity compared with parental vMCF-7^Raf-1^ 1GX cells, supporting the role of NOTCH stemness signaling in inducing ALDH1 activity and an increased metastatic behavior [50]. Next, we defined whether inhibition of self-renewal capacity and tumor stemness was functionally linked to loss of invasive capacity. An in vitro real-time invasion assay showed that abrogation of NOTCH3 expression significantly inhibited the invasive capacity of vMCF-7^Raf-1^ 1GX cells, indicating that NOTCH3 signaling pathway is necessary to promote a more aggressive phenotype. Because we have previously demonstrated the causative role of aberrant AURKA activity in driving the development of breast cancer metastases [29], we aimed to establish whether NOTCH3 expression was required to mediate AURKA-induced highly invasive capacity of vMCF-7^∆Raf1^ 1GX cells. Forced expression of AURKA in vMCF-7^∆Raf1^ 1GX cells increased NOTCH3 expression and their in vitro invasive capacity. Conversely, AURKA overexpression in vMCF-7^Raf-1^ 1GX^CRISPR-NOTCH3^ cells failed to restore a highly invasive phenotype, demonstrating that NOTCH3 expression is required to mediate AURKA-induced high metastatic potential. Moreover, these results highlight a novel mechanistic linkage between AURKA and NOTCH3 oncogenic pathways that is critical to development of a fully metastatic phenotype in breast cancer cells. To define in a different breast cancer model whether increased expression of NOTCH3 was restricted to metastatic cancer cells, we employed MDA-MB-231 TNBC cells that exhibit a CD44^high^/CD24^low^ basal-like phenotype and elevated endogenous MAPK activity [34, 52]. Significantly, the percentage of ex vivo MDA-MB-231 cells isolated from lung metastases (MDA-MB-231 LM) expressing NOTCH3 was higher than matching MDA-MB-231 parental cells, suggesting that NOTCH3 signaling is also required for the metastatic seeding and growth of TNBC cells. These results are in agreement with a recent study that demonstrated the role of the NOTCH3 signaling pathway in promoting the growth of basal-like breast cancer cells [51–53]. Complementary to our vMCF-7^Raf-1^ model, targeting of NOTCH3 impaired the in vitro invasive capacity of MDA-MB-231 LM cells, demonstrating the key role of NOTCH3 expression in promoting the TNBC highly invasive phenotype. Next, we aimed to determine whether inhibition of NOTCH signaling decreased the metastatic capacity of MDA-MB-231 LM cells. In vitro treatment of MDA-MB-231 LM cells with the pan-NOTCH inhibitor LY-411575 resulted in the inhibition of cancer cell seeding and onset of experimental lung metastases, demonstrating that NOTCH pharmacologic targeting interferes with late stages of the invasion-metastasis cascade in NOTCH3-expressing breast cancer cells. Importantly, MDA-MB-231 LM and matching parental cells expressed nominal levels of NOTCH1 and NOTCH2, suggesting that LY411575-mediated inhibition of cancer cell seeding and metastatic growth was primarily linked to inhibition of the NOTCH3 signaling pathway.

Owing to the limited translatability of established cancer cells, and to corroborate the central role of NOTCH3 in driving a metastatic phenotype in clinically relevant models, we established unique TNBC cells (TNBC-M25) isolated from a patient-derived brain metastasis xenograft model. TNBC-M25 cells showed high expression of phospho-AURKA and NOTCH3, whereas NOTCH1 and NOTCH2 levels were low, suggesting that the AURKA/NOTCH3 oncogenic axis plays a major role in promoting their metastatic phenotype. To test this hypothesis, we reduced NOTCH3 expression by lenti-shRNAs in TNBC-M25 cells. Reduction of NOTCH3 expression impaired self-renewal capacity, resulting in a significant shrinkage of TNBC-M25 MPS, confirming the essential role of the NOTCH3 signaling pathway in promoting tumor stemness. Moreover, inhibition of NOTCH3 expression also reduced in vitro the invasiveness of TNBC-M25 cells. These results validated our findings in vMCF-7^∆Raf1^ 1GX cells that the NOTCH3 signaling pathway is downstream of AURKA and is required to promote breast cancer cells’ aggressiveness.

Finally, our findings in established and patient-derived breast cancer cells led us to analyze the correlation between aberrant expression of NOTCH3 and the overall survival of patients with claudin-low breast tumors using copy number aberrations, somatic mutations, and gene expression data derived from the METABRIC study [30]. We selected claudin-low breast tumors because they represent a molecular subtype of breast cancer with high metastatic proclivity and poor outcome originally identified by gene expression profiling [40]. The claudin-low subgroup analyzed represented a cluster of 125 patients characterized by 112 TNBC and 13 ER^−^/PR^−^/HER2^+^ specimens. Our analysis showed that aberrant NOTCH3 expression was significantly associated with decreased overall patient survival, supporting the pivotal role of NOTCH3 oncogenic pathway in promoting breast cancer progression.

## Conclusions

**Fig. 11:**
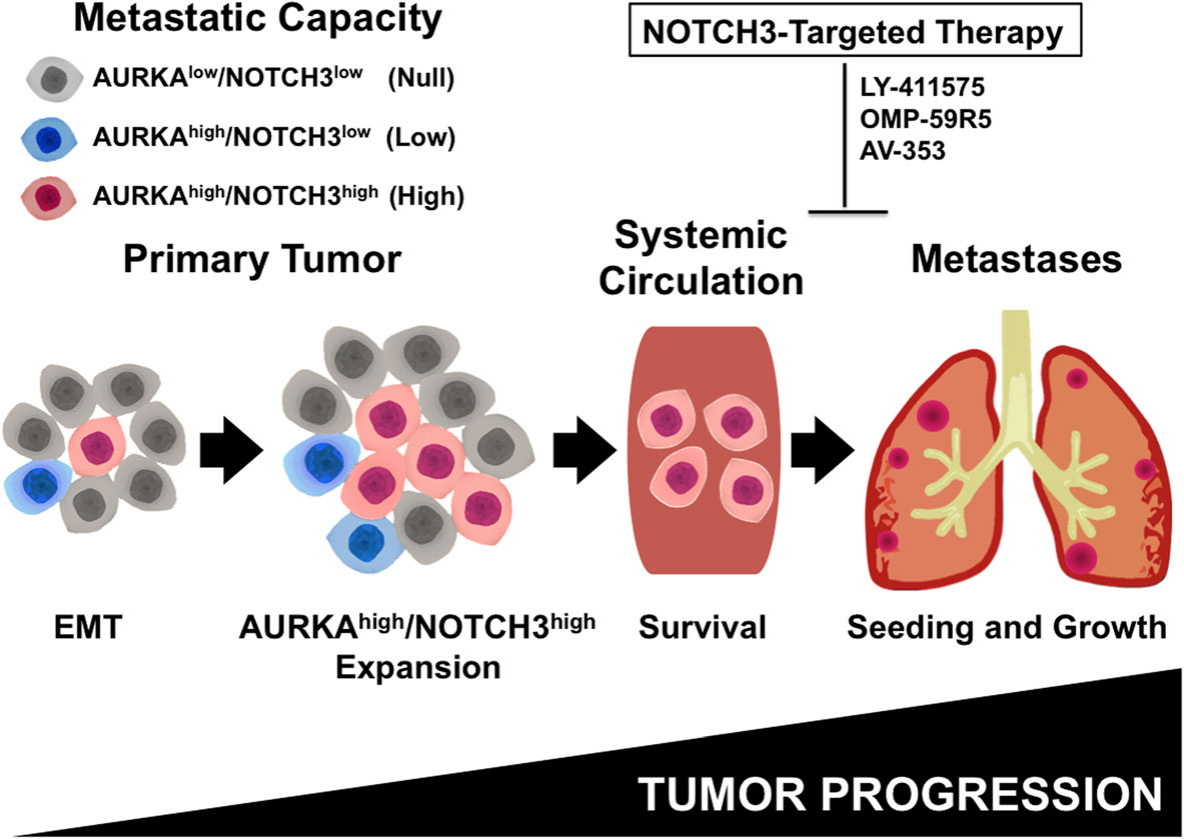
AURKA^high^/NOTCH3^high^ breast tumor metastasis-initiating cells (BT-MICs) promote cancer cell seeding and metastatic growth. Primary breast tumors show heterogeneous subclones where the majority of cancer cells exhibit an AURKA^low^/NOTCH3^low^ phenotype with low invasive capacity. Increased expression and activity of Aurora kinase A (AURKA) during tumor growth will induce epithelial-mesenchymal transition (EMT) and the genesis of AURKA^high^/NOTCH3^low^ BTIC subclones with increased invasive capacity but incapable of giving rise to distant metastases. Gain of NOTCH3 expression in AURKA^high^/NOTCH3^high^ BTICs will lead to the clonal expansion of AURKA^high^/NOTCH3^high^ BT-MICs that will successfully complete the invasion-metastasis cascade. Pharmacologic inhibition of NOTCH3 signaling with either pan-NOTCH inhibitors or humanized monoclonal antibodies will halt AURKA^high^/NOTCH3^high^ BT-MICs seeding to secondary organs and metastatic growth

On the basis of our previous studies [5, 29, 34] and the results presented here, we propose a novel model of breast cancer progression. Primary breast tumors are comprised of heterogeneous subclones where bulk cancer cells exhibit a nontumorigenic AURKA^low^/NOTCH3^low^ phenotype that lacks EMT, stemness activity, and invasive and metastatic capacity. Increased expression and activation of AURKA will induce EMT and the genesis of AURKA^high^/NOTCH3^low^ BTIC subclones, and it is unlikely that these subclones will be competent to complete the invasion-metastasis cascade, owing to their limitations in high self-renewal/invasive capacity and seeding in a new microenvironment. Gain of aberrant activation of NOTCH3 oncogenic pathway among BTICs will lead to the clonal expansion of AURKA^high^/NOTCH3^high^ BT-MICs that have acquired strong stemness properties and the capacity to successfully complete the invasion-metastasis cascade. Conversely, pharmacologic inhibition of NOTCH3 signaling inhibits AURKA^high^/NOTCH3^high^ BT-MIC seeding to secondary organs and metastatic growth (Fig. 11). Because we identified a novel cross-talk between AURKA and NOTCH3 oncogenic pathways in promoting breast cancer progression, we speculate that dual-targeted therapy with selective inhibitors of AURKA and NOTCH3 could also represent a novel stemness-targeted therapeutic strategy to successfully eradicate BT-MICs, particularly for the clinical management of highly aggressive TNBCs that currently lack effective U.S. Food and Drug Administration-approved targeted therapies.

## Additional files

Additional file 1: **Figure S1.** Expression of CD24 luminal marker in MPS derived from variant vMCF-7^∆Raf1^ and vMCF-7^∆Raf1^ 1GX-M cells. **a** Immunofluorescence analysis showing representative images of vMCF-7^∆Raf1^ and vMCF-7^∆Raf1^ 1GX-M MPS stained in *red* with a CD24 monoclonal antibody. Nuclei were stained in *blue* with 4′,6-diamidino-2-phenylindole (DAPI). **b** Graph showing the average number of CD24-expressing cells from three independent experiments (± SD). (TIFF 6168 kb)

Additional file 2: **Figure S2.** Transcriptomic characterization of metastatic breast cancer cells. **a** Comparative global gene array analysis between CD24^−/low^ (isolated by FACS sorting from vMCF-7^Raf-1^ 1GX cells) and vMCF-7^Raf-1^ 1GX-M MPS. **b** In silico comparative functional enrichment analysis between CD24^−/low^ (isolated from vMCF-7^Raf-1^ 1GX cells) and vMCF-7^Raf-1^ 1GX-M MPS identified 59 genes involved in nuclear reprograming. (TIFF 6168 kb)

Additional file 3: **Figure S3.** Expression of genes identified in NOTCH3 metastatic network. Graphs showing the average expression values in sample replicates (from two independent experiments ± SD) for each gene represented in the NOTCH3 metastatic network. (TIFF 6168 kb)

Additional file 4: **Figure S4.** CRISPR-NOTCH3 breast cancer cells. **a** NOTCH3 gene knockout using CRISPR/Cas9. Lightning bolt symbols indicate the targeted gene double-stranded break (DSB) sites for different sgRNAs F1 and R2. *Horizontal arrows* show the PCR primers designed at different chromosomal sites to identify deletions. **b** A PCR product of ~ 650-bp size is amplified upon a successful double-hit by SRISPR/Cas9 system. **c** Secondary screening using internal primers. Internal primers were used to screen for clones with efficient gene knockout. Clone 416 was selected for further verification by immunoblot assay (Fig. 4a). (TIFF 6168 kb)

Additional file 5: **Figure S5.** NOTCH1 and NOTCH2 expression in TNBC cells. **a** Immunofluorescence analysis showing representative images of MDA-MB-231 and MDA-MB-231 LM TNBC cells stained in *green* with NOTCH1 and NOTCH2 polyclonal antibodies. Nuclei were stained in *blue* with DAPI. **b** Graphs showing the average number of NOTCH1- and NOTCH2-expressing cells from three independent experiments (± SD). (TIFF 6168 kb)

Additional file 6: **Figure S6.** NOTCH1 and NOTCH2 expression in patient-derived TNBC cells. **a** Immunoblot assay showing NOTCH1 and NOTCH2 expression in MDA-MB-231 and patient-derived TNBC-M25 cells. **b** Densitometric analysis showing the percentage of NOTCH1 and NOTCH2 protein levels in TNBC-M25 cells relative to MDA-MB-231 cells. Graph showing the average from three independent experiments (± SD). (TIFF 6168 kb)
