# Supplementary figures and images for: NOTCH3 expression is linked to breast cancer seeding and distant metastasis
Source: Breast Cancer Res. 2018 Sep 4;20:105. doi: 10.1186/s13058-018-1020-0 (PMC6123953; doi:10.1186/s13058-018-1020-0)

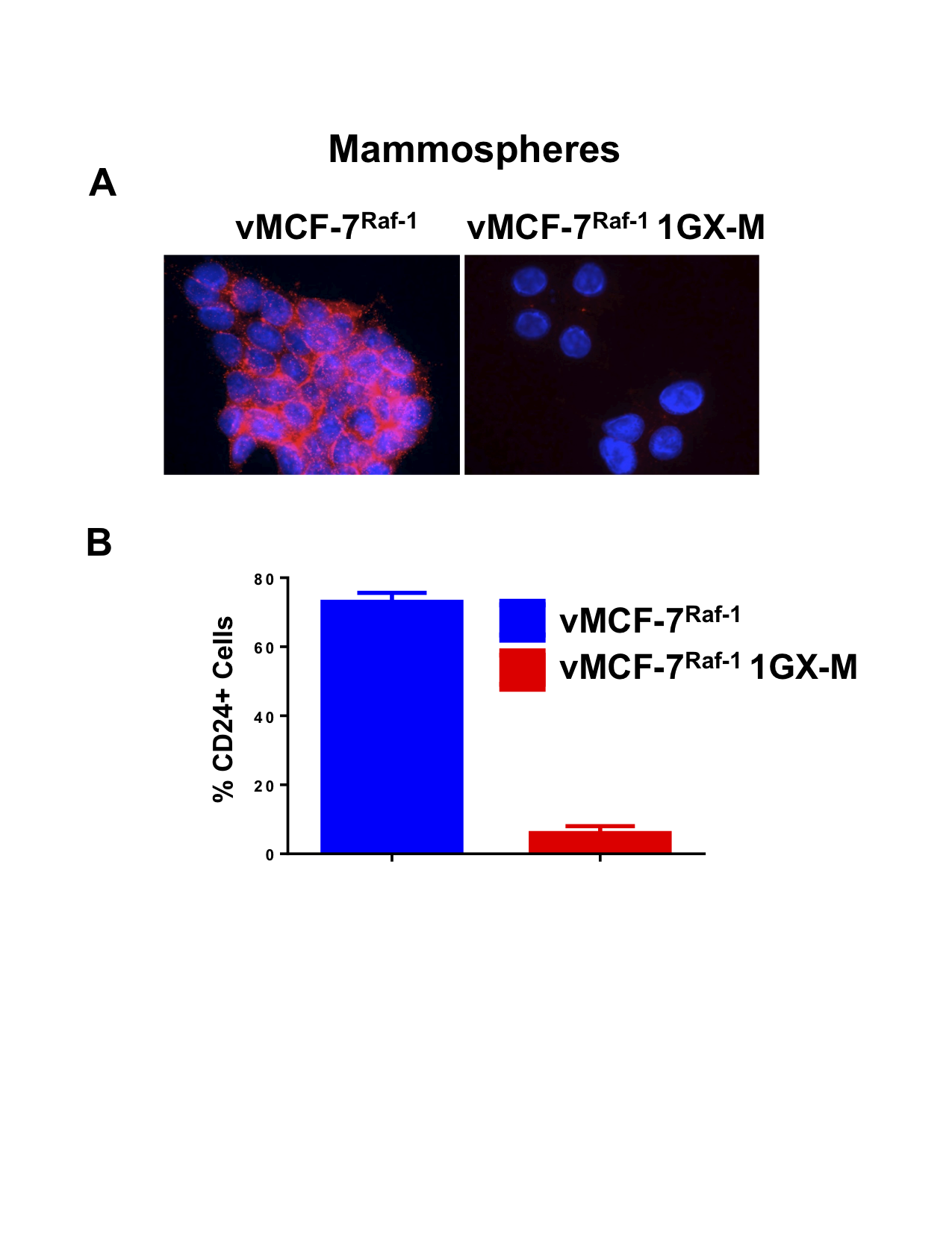

Supplement: Supplementary file 1 — Figure S1. Expression of CD24 luminal marker in MPS derived from variant vMCF-7∆Raf1 and vMCF-7∆Raf1 1GX-M cells. a Immunofluorescence analysis showing representative images of vMCF-7∆Raf1 and vMCF-7∆Raf1 1GX-M MPS stained in red with a CD24 monoclonal antibody. Nuclei were stained in blue with 4′,6-diamidino-2-phenylindole (DAPI). b Graph showing the average number of CD24-expressing cells from three independent experiments (± SD). (TIFF 6168 kb) [file 13058_2018_1020_MOESM1_ESM.tiff]

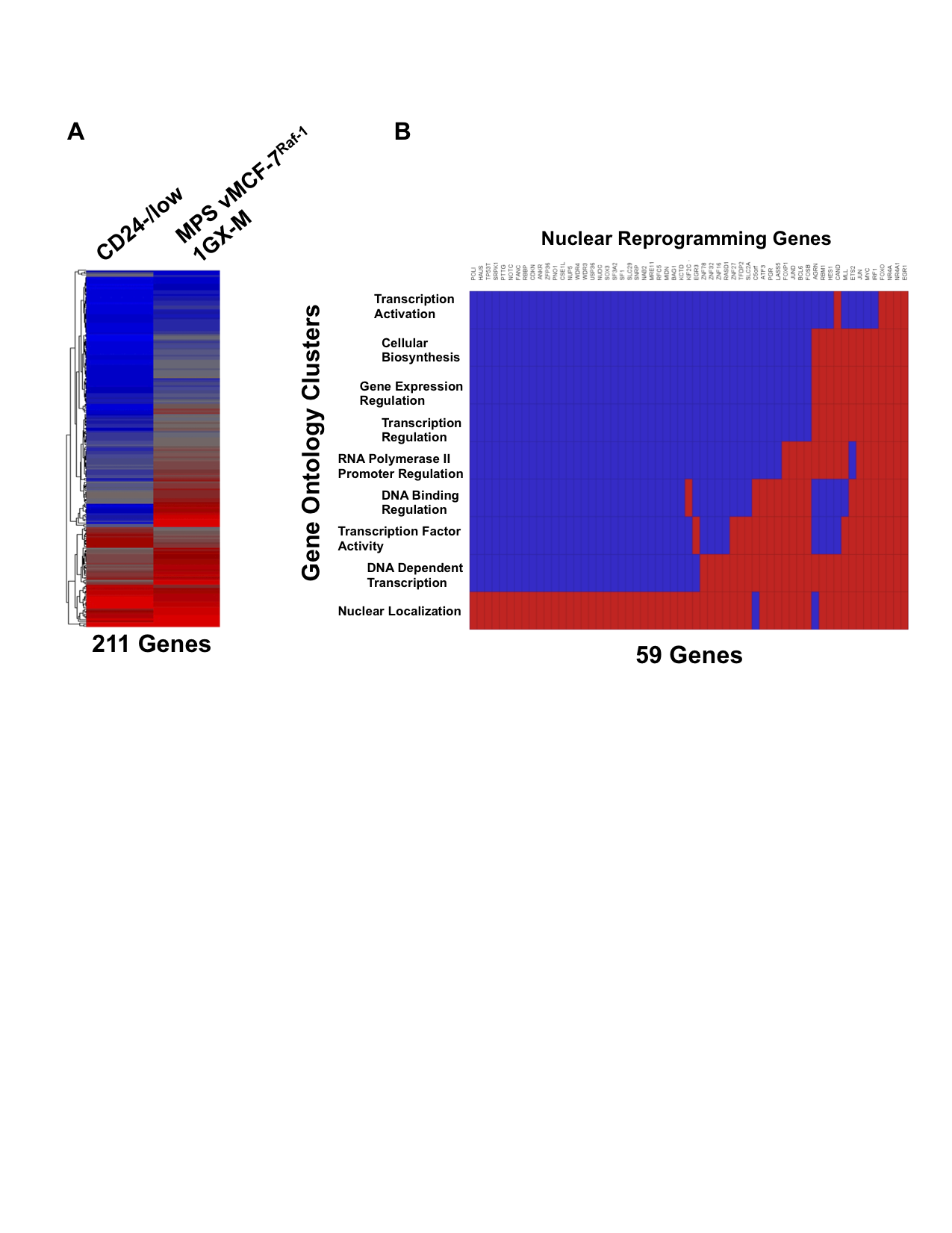

Supplement: Supplementary file 2 — Figure S2. Transcriptomic characterization of metastatic breast cancer cells. a Comparative global gene array analysis between CD24−/low (isolated by FACS sorting from vMCF-7Raf-1 1GX cells) and vMCF-7Raf-1 1GX-M MPS. b In silico comparative functional enrichment analysis between CD24−/low (isolated from vMCF-7Raf-1 1GX cells) and vMCF-7Raf-1 1GX-M MPS identified 59 genes involved in nuclear reprograming. (TIFF 6168 kb) [file 13058_2018_1020_MOESM2_ESM.tiff]

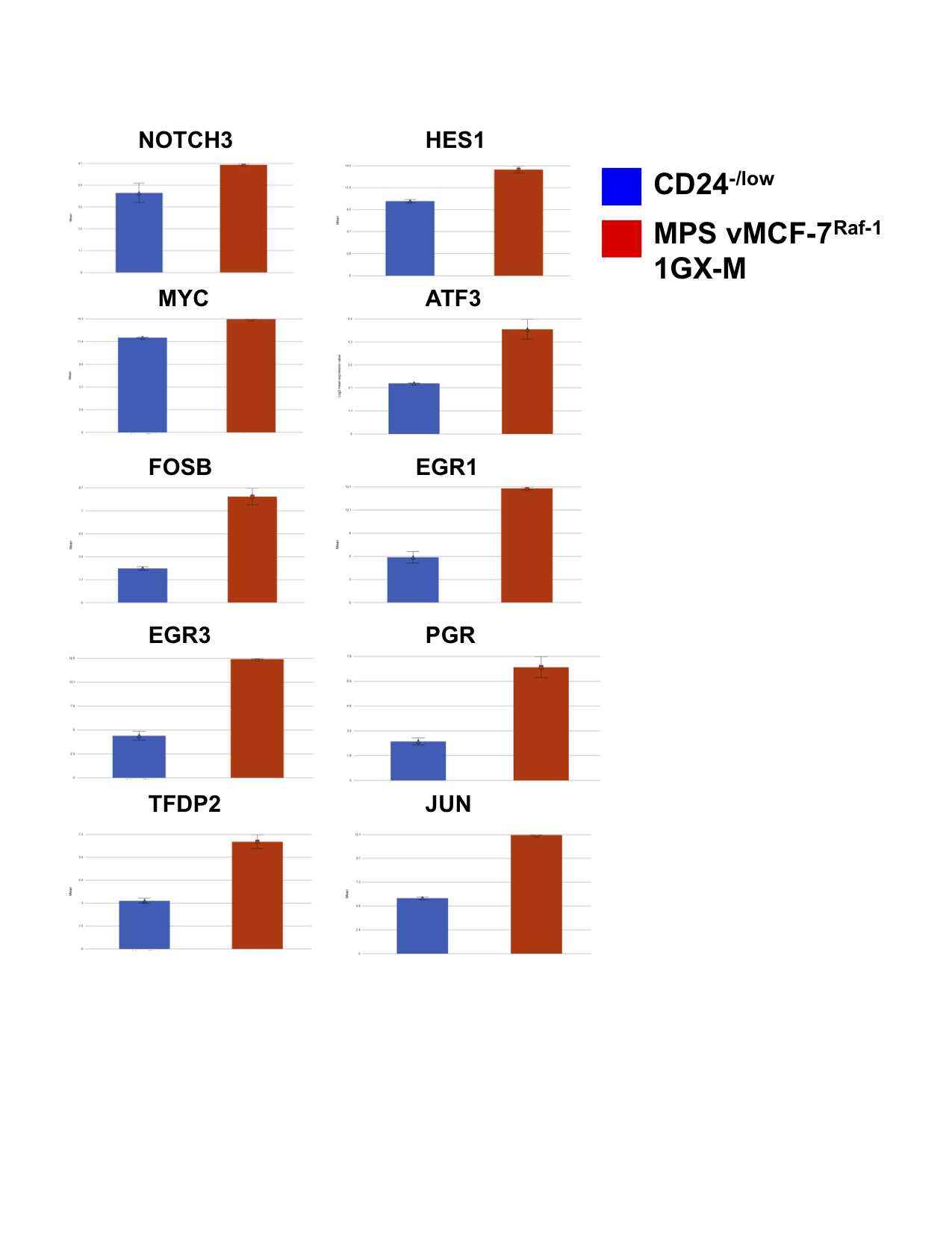

Supplement: Supplementary file 3 — Figure S3. Expression of genes identified in NOTCH3 metastatic network. Graphs showing the average expression values in sample replicates (from two independent experiments ± SD) for each gene represented in the NOTCH3 metastatic network. (TIFF 6168 kb) [file 13058_2018_1020_MOESM3_ESM.tiff]

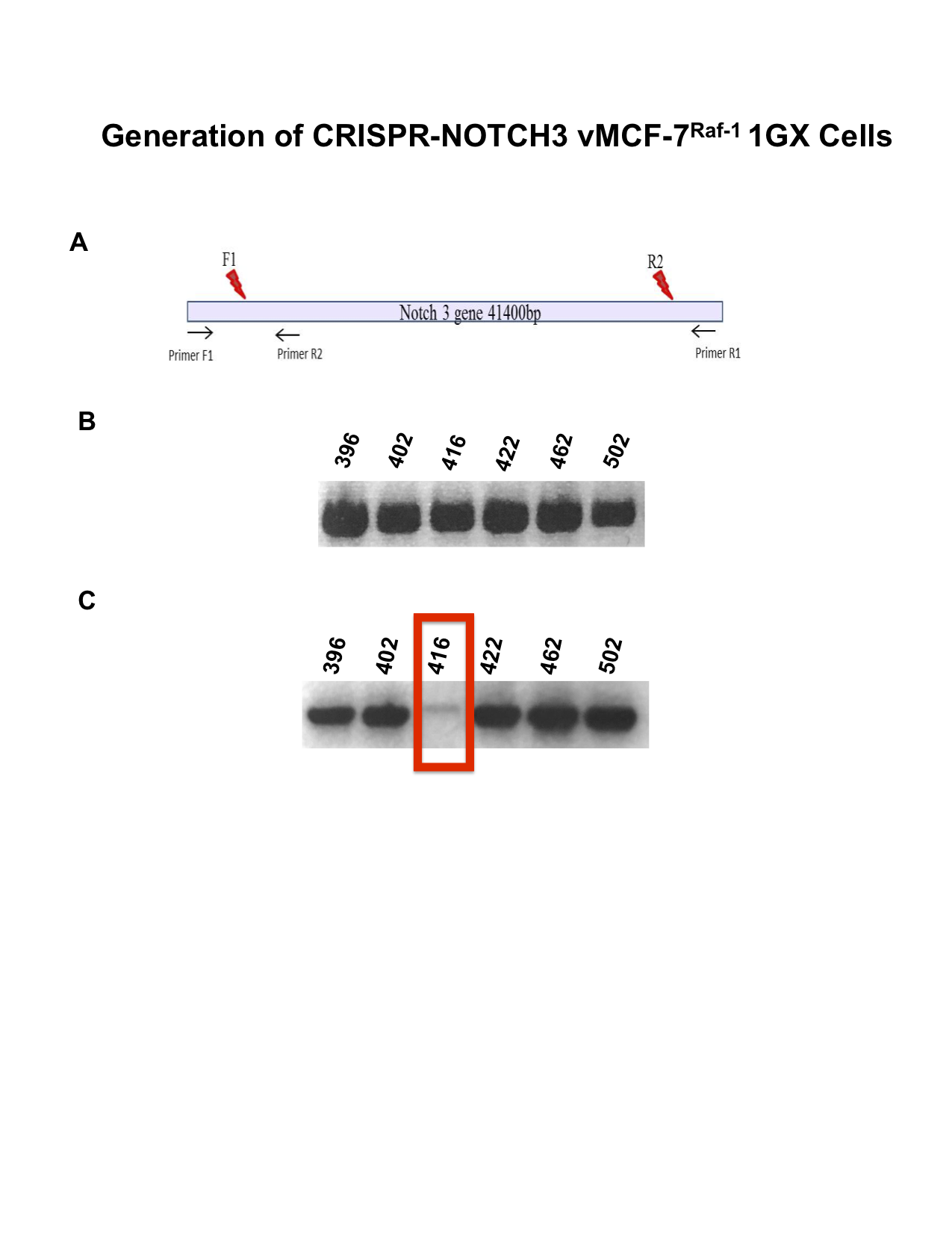

Supplement: Supplementary file 4 — Figure S4. CRISPR-NOTCH3 breast cancer cells. a NOTCH3 gene knockout using CRISPR/Cas9. Lightning bolt symbols indicate the targeted gene double-stranded break (DSB) sites for different sgRNAs F1 and R2. Horizontal arrows show the PCR primers designed at different chromosomal sites to identify deletions. b A PCR product of ~ 650-bp size is amplified upon a successful double-hit by SRISPR/Cas9 system. c Secondary screening using internal primers. Internal primers were used to screen for clones with efficient gene knockout. Clone 416 was selected for further verification by immunoblot assay (Fig. 4a). (TIFF 6168 kb) [file 13058_2018_1020_MOESM4_ESM.tiff]

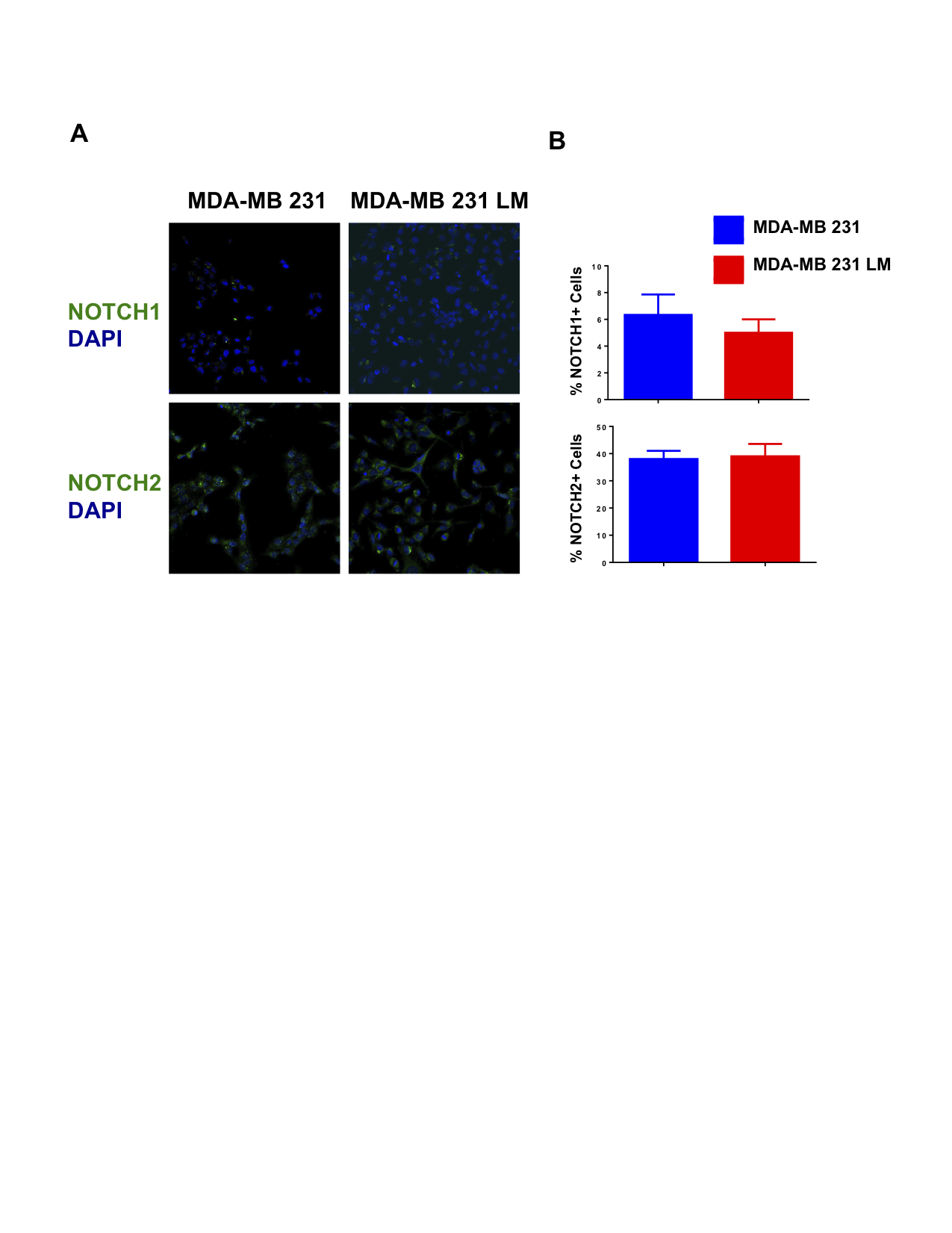

Supplement: Supplementary file 5 — Figure S5. NOTCH1 and NOTCH2 expression in TNBC cells. a Immunofluorescence analysis showing representative images of MDA-MB-231 and MDA-MB-231 LM TNBC cells stained in green with NOTCH1 and NOTCH2 polyclonal antibodies. Nuclei were stained in blue with DAPI. b Graphs showing the average number of NOTCH1- and NOTCH2-expressing cells from three independent experiments (± SD). (TIFF 6168 kb) [file 13058_2018_1020_MOESM5_ESM.tiff]

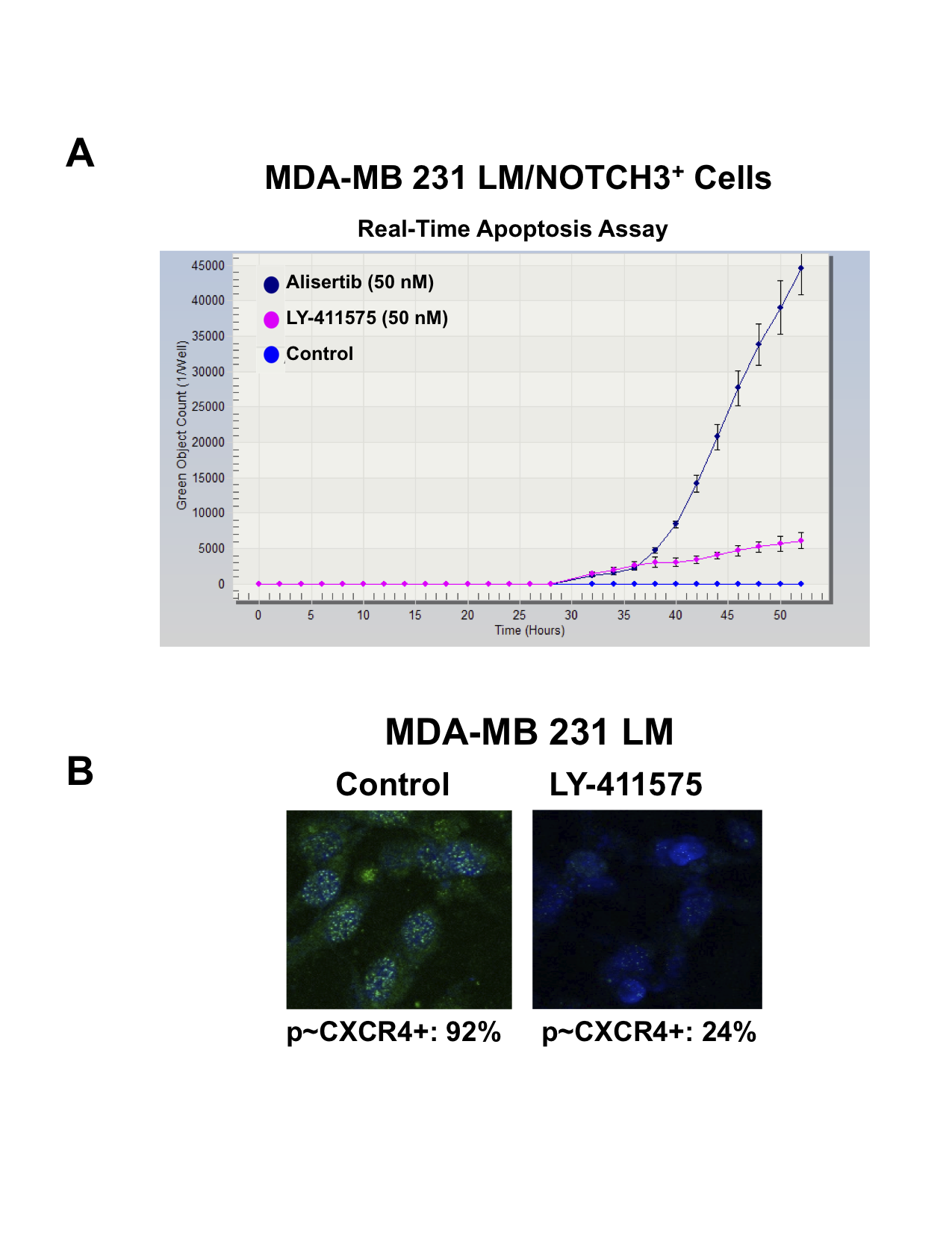

Supplement: Supplementary file 6 — Figure S6. NOTCH1 and NOTCH2 expression in patient-derived TNBC cells. a Immunoblot assay showing NOTCH1 and NOTCH2 expression in MDA-MB-231 and patient-derived TNBC-M25 cells. b Densitometric analysis showing the percentage of NOTCH1 and NOTCH2 protein levels in TNBC-M25 cells relative to MDA-MB-231 cells. Graph showing the average from three independent experiments (± SD). (TIFF 6168 kb) [file 13058_2018_1020_MOESM6_ESM.tiff]
